# Identification of a combined apoptosis and hypoxia gene signature for predicting prognosis and immune infiltration in breast cancer

**DOI:** 10.1002/cam4.4755

**Published:** 2022-04-20

**Authors:** Xueting Ren, Hanxiao Cui, Jianhua Wu, Ruina Zhou, Nan Wang, Dandan Liu, Xin Xie, Hao Zhang, Di Liu, Xiaobin Ma, Chengxue Dang, Huafeng Kang, Shuai Lin

**Affiliations:** ^1^ Department of Oncology The Second Affiliated Hospital of Xi'an Jiaotong University, Xi'an Shaanxi China; ^2^ Department of Surgical Oncology The First Affiliated Hospital of Xi'an Jiaotong University, Xi'an Shaanxi China

**Keywords:** breast cancer, cancer genetics, immunology, microenvironment, prognosis, risk model

## Abstract

**Background:**

Breast cancer (BC) is the most common malignant tumor worldwide. Apoptosis and hypoxia are involved in the progression of BC, but reliable biomarkers for these have not been developed. We hope to explore a gene signature that combined apoptosis and hypoxia‐related genes (AHGs) to predict BC prognosis and immune infiltration.

**Methods:**

We collected the mRNA expression profiles and clinical data information of BC patients from The Cancer Genome Atlas database. The gene signature based on AHGs was constructed using the univariate Cox regression, least absolute shrinkage and selection operator, and multivariate Cox regression analysis. The associations between risk scores, immune infiltration, and immune checkpoint gene expression were studied using single‐sample gene set enrichment analysis. Besides, gene signature and independent clinicopathological characteristics were combined to establish a nomogram. Finally, Gene Ontology (GO) and Kyoto Encyclopedia of Genes and Genomes (KEGG) were performed on the potential functions of AHGs.

**Results:**

We identified a 16‐AHG signature (AGPAT1, BTBD6, EIF4EBP1, ERRFI1, FAM114A1, GRIP1, IRF2, JAK1, MAP2K6, MCTS1, NFKBIA, NFKBIZ, NUP43, PGK1, RCL1, and SGCE) that could independently predict BC prognosis. The median score of the risk model divided the patients into two subgroups. By contrast, patients in the high‐risk group had poorer prognosis, less abundance of immune cell infiltration, and expression of immune checkpoint genes. The gene signature and nomogram had good predictive effects on the overall survival of BC patients. GO and KEGG analyses revealed that the differential expression of AHGs may be closely related to tumor immunity.

**Conclusion:**

We established and verified a 16‐AHG BC signature which may help predict prognosis, assess potential immunotherapy benefits, and provide inspiration for future research on the functions and mechanisms of AHGs in BC.

## Graphical abstract

We identified a 16‐AHG signature (AGPAT1, BTBD6, EIF4EBP1, ERRFI1, FAM114A1, GRIP1, IRF2, JAK1, MAP2K6, MCTS1, NFKBIA, NFKBIZ, NUP43, PGK1, RCL1, SGCE) that could independently predict BC prognosis. The median score of the risk model divided the patients into two subgroups. By contrast, patients in the high‐risk group had poorer prognosis, less abundance of immune cell infiltration, and expression of immune checkpoint genes. The gene signature and nomogram had good predictive effects on the overall survival of BC patients. GO and KEGG analyses revealed that the differential expression of AHGs may be may be closely related to tumor immunity.

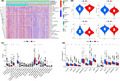

## 1 INTRODUCTION

The incidence of breast cancer (BC) ranks first in the world, and its global burden is still increasing.^1^, ^2^ It is estimated that by 2022, there will be 287,850 new cases of female breast cancer and 43,250 deaths in the United States alone.^3^ Existing therapeutic modalities such as chemotherapy, surgery, radiotherapy, endocrine therapy, targeted therapy, and immunotherapy have been widely used in clinical practice and have made significant progress in recent years. However, BC still has a high recurrence and mortality rate.^4^ Identification of high‐risk patients to improve treatment accuracy is indispensable for improving prognosis. Therefore, developing valuable BC biomarkers is paramount for patient selection and therapy response prediction.

Cell death has various forms such as apoptosis, necrosis, pyrosis, oncosis, and autophagy, which have their own characteristics.^5^ In recent years, the understanding of the various forms of cell death has deepened. Interrupting cell death for cancer treatment has been widely investigated. Apoptosis is a type of programmed cell death that contributes to the control of cell proliferation, elimination of harmful and unessential cells in vivo, and maintenance of tissue homeostasis in multicellular organisms.^6^ Therefore, apoptotic signals help protect genome integrity and maintain organism integrity.^7^ Evasion of apoptosis is considered a hallmark of cancer. Inhibition of apoptotic pathways can enhance the viability of cancer cells, thereby promoting their uncontrolled proliferation.^8^ Hypoxia is a typical factor of almost all solid tumor microenvironments and induces apoptosis.^9^ Hypoxia inducible factor‐1 (HIF‐1) is indispensable in regulating this process.^10^ It can increase the expression of pro‐apoptotic proteins (such as BNIP3) and initiate hypoxia‐induced apoptosis, or regulate BAX, BAK, and other proteins to induce apoptosis by stabilizing protein products of tumor suppressor gene p53.^11^, ^12^ Hence, apoptosis and hypoxia are closely related and interact with each other in the process of tumorigenesis and development.

Increasing studies have shown that the immune system is integral to the occurrence and development of BC, and immunotherapy may ameliorate the clinical results of BC.^13^, ^14^ The clinical activity and safety of immunotherapy have been preliminarily confirmed in early BC vaccine trials.^15^, ^16^ New immune regulation strategies, such as those targeting myeloid suppressor cells and regulatory T cells, have also received widespread attention.^17^, ^18^ Notably, blocking immune checkpoints has shown potential in the treatment of BC. Immunotherapy targeting programmed cell death‐1/programmed death ligand‐1 (PD‐1/PD‐L1) has a survival benefit in some patients with metastatic triple‐negative BC (TNBC).^13^ At present, the main challenges of immunotherapy are still identifying biomarkers that can predict the potential response to immunotherapy, as well as selecting appropriate target populations. Some studies have confirmed that tumor cells directly participate in immune escape by acquiring apoptosis resistance.^19^ Apoptosis resistance may not only be related to tumorigenesis and chemotherapy resistance, but also affect immune monitoring and immunotherapy. Besides hypoxia, stress causes immunosuppression by controlling angiogenesis, as well as by promoting immunosuppression and tumor resistance.^20^

In evaluating the relationships between apoptosis, hypoxia, and the immune system in the tumor microenvironment (TME), we identified a gene signature that combined apoptosis and hypoxia‐related genes (AHGs) to evaluate the prognosis of BC, supported by The Cancer Genome Atlas (TCGA) database. Moreover, assessing immune infiltration by risk score was helpful to select the appropriate population for immunotherapy. In addition, we constructed a nomogram by integrating the risk model with several clinicopathological features to quantitatively predict the survival of BC patients.

## 2 MATERIALS AND METHODS

### 2.1 Collection and preparation of data

We collected RNA sequencing profiles of 1109 BC samples and 113 healthy controls from the TCGA database (https://portal.gdc.cancer.gov/). These gene expression data were then formatted into fragments per kilobase of transcript per million mapped reads (FPKM) and normalized by log_2_(FPKM+1) in the gene expression comparative analysis. In addition, we collected detailed clinical data of these BC cases, including age, survival time and status, TNM stage, pathological stage, and expression status of the estrogen receptor (ER), progesterone receptor (PR), and human epidermal growth factor receptor 2 (HER2). Moreover, another external verification cohort of 1052 BC cases from the International Cancer Genome Consortium (ICGC) database (https://dcc.icgc.org/projects/BRCA‐US) was obtained to verify our findings. Figure S1 displays the analysis procedures of this study.

### 2.2 Identification of differentially expressed apoptosis and hypoxia‐related genes

Gene set enrichment analysis (GSEA) is a tool for analyzing the differential expression of annotated genes or gene sets and interpreting the results in the biological processes involved.^21^ The molecular signatures database (MSigDB, https://www.gsea‐msigdb.org/gsea/msigdb/index.jsp) was originally developed for GSEA and other similar approaches and has been one of the biggest and most influential libraries of gene sets. The most recent version of MSigDB has nine collections (H and C1‐C8), including hallmark gene sets (H), positional gene sets (C1), curated gene sets (C2), regulatory target gene sets (C3), computational gene sets (C4), ontology gene sets (C5), oncogenic signature gene sets (C6), immunologic signature gene sets (C7), and cell type signature gene sets (C8).^22^, ^23^ Based on the MSigDB, we screened apoptosis‐ and hypoxia‐related gene sets for subsequent analyses.

The list of 29 apoptosis‐ and 49 hypoxia‐related gene sets selected from the MSigDB for GSEA included 2556 and 4610 genes, respectively. The “limma” R package was used to identify differentially expressed genes in these sets after normalization.

### 2.3 Construction and verification of the gene signature

To begin, we conducted univariate Cox regression analysis on differentially expressed genes (DEGs) and screened for genes that were meaningfully linked to overall survival (OS) in BC. Then, to reduce the risk of overfitting, we constructed a penalty function and used the least absolute shrinkage and selection operator (LASSO) regression to obtain a more accurate signature. Lastly, multivariate Cox regression analysis was performed to examine the genes acquired in the previous stage, and the final genes were utilized to create an independent gene prediction signature. The following equation was used to determine the gene signature's risk score: Risk score = h (t, X) = h_0_(t) × e^Ʃ (coefi * Expri)^. In this formula, Expri represents gene expression, while h_0_(t) and coefi represent constant and coefficient obtained in multivariate Cox regression analysis, respectively. Each patient's risk score was determined in both the TCGC and ICGC cohorts, and the high‐ and low‐risk groups were separated based on the median risk score. The survival difference between these two subgroups was assessed using the Kaplan–Meier (KM) survival analysis and the log‐rank test. Likewise, receiver operating characteristic (ROC) analysis was conducted to further evaluate the prognostic signature's accuracy. These studies used the R packages “Survminer” and “survivalROC”.

### 2.4 Associations between risk score and immune infiltration profiles and immune checkpoint gene expression in BC

We assessed the tumor purity and immune, stromal, and estimate scores of high‐ and low‐risk groups using the R package “estimate” and unsupervised consensus cluster analysis, and then estimated the distribution of stromal and immune cells in tumor tissues using the “estimate” R package.^24^, ^25^ The CIBERSORT technique was used in our work to determine the relative percentage of 22 immune cells in each tumor tissue sample, using the LM22 signature matrix to run the algorithm under 1000 permutations.^26^, ^27^ Next, we explored the relationship between gene signature's risk scores and immune scores, infiltration of immune cells and immune‐related pathways, and expression of immune checkpoints based on the single‐sample GSEA (ssGSEA) of the “GSVA” R package.^28^

### 2.5 Establishment and assessment of a nomogram based on the combined apoptosis and hypoxia gene signature

A nomogram was created to quantitatively estimate the OS in BC patients by incorporating the combined apoptosis and hypoxia gene signature with clinicopathological features that can independently predict prognosis. Cox regression analysis assigned a certain score to each variable in the nomogram to predict the 3‐, and 5‐year survival rates. Scores were negatively correlated with prognosis. Moreover, Harrell's concordance index (C‐index), KM survival analysis, the area under the ROC curve (AUC), and calibration curves were employed to assess the nomogram's prediction performance. The higher the C‐index, the stronger the prediction power of the nomogram. The nomogram‐predicted survival rates and observed survival rates were plotted on the x‐ and y‐axes of the calibration curves, with the 45‐degree line representing the best prediction. The nomogram was evaluated by bootstrap method with 1000 heavy samples.

### 2.6 Functional enrichment analysis

The DEGs between high‐ and low‐risk groups were identified by the cutoff values of |log_2_ fold change (FC)| > 1 and false discovery rate (FDR) < 0.05. The “limma” and “clusterProfiler” R package was used to conduct Gene Ontology (GO) and Kyoto Encyclopedia of Genes and Genomes (KEGG) pathway enrichment analysis to explore the underlying effect of the AHGs on the development of BC.^29^ Biological process (BP), cellular component (CC), and molecular function (MF) were the three categories examined in the GO analysis.^30^ A relevant threshold for evaluating functional pathways was set at *p* < 0.05.

### 2.7 Statistical analysis

All statistical analysis and charts were obtained using R (version 4.0.2) and Excel (Microsoft Corporation, California). To evaluate OS differences comparing high‐ and low‐risk groups, KM curves, and log‐rank tests were utilized. The hazard ratio (HR) and 95 percent confidence interval (CI) of prognostic variables were calculated in both univariate and multivariate Cox regression models to select independent prognostic factors. The chi‐square test and Mann–Whitney *U* test were applied to assess the correlations between risk scores and clinicopathological variables as well as immune cells or pathways. The two‐tailed tests were given a statistical significance of *p* < 0.05.

## 3 RESULTS

### 3.1 Characteristics of BCpatients included in the study

Due to the missing values of OS and survival status, and the situation that one patient may has multiple samples, only 1090 BC patients with transcriptome profiles and detailed clinicopathological parameters were selected for subsequent analysis from the TCGA database (Table 1). The average age of patients in the TCGA cohort was 58.6 years old, with an average follow‐up of 3.4 years. Among them, 800 patients were at AJCC stage I–II (74.98%), and 267 patients were at stage III–IV (25.02%). We also included 989 BC patients from the ICGC (BRCA‐US) cohort to validate our risk prognostic model. The average age of the patients in the validation group was 58.4 years, with a mean follow‐up of 2.4 years, according to their survival data.

**TABLE 1:** Clinical pathological parameters of patients with BC

Clinical pathological parameters	*N*	%
Age(years)		
<=65	771	70.73
>65	319	29.27
Gender		
Female	1078	98.90
Male	12	1.10
T classification		
T1‐T2	910	83.72
T3‐T4	177	16.28
N classification		
N0	514	48.04
N1‐N3	556	51.96
M classification		
M0	907	97.63
M1	22	2.37
Pathological stage		
Stage I‐II	800	74.98
Stage III‐IV	267	25.02
ER status		
Negative	238	22.91
Positive	801	77.09
PR status		
Negative	343	33.08
Positive	694	66.92
HER2 status		
Negative	561	77.49
Positive	163	22.51

### 3.2 Determination of differentially expressed AHGsin BCand normal samples

The expression of genes from 29 apoptosis‐ and 49 hypoxia‐related gene sets was estimated. The results showed that compared with the healthy control samples derived from TCGA database, there were 1805 downregulated and 1932 upregulated AHGs in BC tissues (Table S1).

### 3.3 Construction of the combined gene signature for prognosis prediction in BC

**FIGURE 1:**
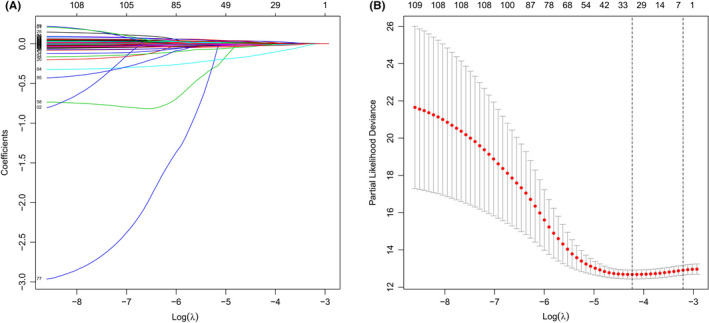
LASSO regression analysis based on differentially expressed genes. (A) Ten‐fold cross‐validation for the coefficients. (B) Parameter selection of the 31 selected AHGs in LASSO regression (λ = −4.2)

First, we obtained 113 prognostic genes (59 upregulated and 54 downregulated) in BC patients by univariate Cox regression analysis (Table S2). Next, LASSO regression showed that the cross‐validation error was the smallest when λ = −4.2, and the corresponding 31 genes entered the multivariate Cox regression analysis (Figure 1A,B). Finally, a 16‐AHG signature was obtained to independently estimate BC patients' prognosis.

**FIGURE 2:**
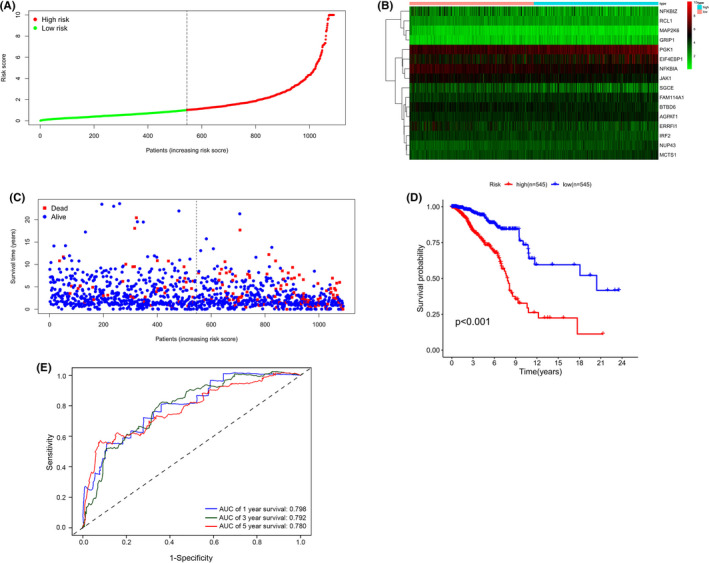
Prognostic analysis of the 16‐AHG risk model in TCGA cohort. (A) Distribution and median of the risk scores. (B) Expression heatmap of 16 AHGs in high‐ and low‐risk groups. (C) Survival status. (D) Kaplan–Meier curves of OS in high‐ and low‐risk patients. (E) Time‐dependent ROC curves of prognostic prediction performance of gene signature

Among them, BTBD6, ERRFI1, IRF2, JAK1, MAP2K6, NFKBIA, NFKBIZ, RCL1, and SGCE were protective genes, which were underexpressed in tumor tissues (Figure S2A–I). However, AGPAT1, EIF4EBP1, FAM114A1, GRIP1, MCTS1, NUP43, and PGK1 were risk genes that were highly expressed in tumor tissues (Figure S2J‐P). On the basis of the median risk score, all patients were split into high‐ and low‐risk groups (Figure 2A). Heatmap revealed the expression patterns of 16 AHGs between two different risk groups. (Figure 2B). According to the scatter plot, the death proportion in the high‐risk group was higher than in the low‐risk group (Figure 2C). The KM survival curve data revealed that the OS of the high‐risk group was significantly poorer (*p* < 0.001, Figure 2D). In the TCGA cohort, the AUCs for 1‐, 3‐, and 5‐year OS were 0.798, 0.792, and 0.780, respectively. This suggests that this 16‐AHG signature may robustly assesss the BC patients' prognosis (Figure 2E).

### 3.4 Validation of the 16‐AHGin an ICGCcohort

To verify the ability of the 16‐AHG signature to predict BC prognosis, we selected an external cohort from ICGC. The risk model estimated the risk scores of all selected patients and classified them as high‐risk (*n* = 495) or low‐risk (*n* = 494) patients, respectively. Survival analysis showed that the survival rates of the two risk groups were markedly different (Figure S3A). The AUCs of the 1‐, 3‐, and 5‐year OS of the gene signature in the ICGC validation cohort respectively were 0.841, 0.814, and 0.811 (Figure S3B).

### 3.5 Relationship between risk score and clinicopathological parameters

**FIGURE 3:**
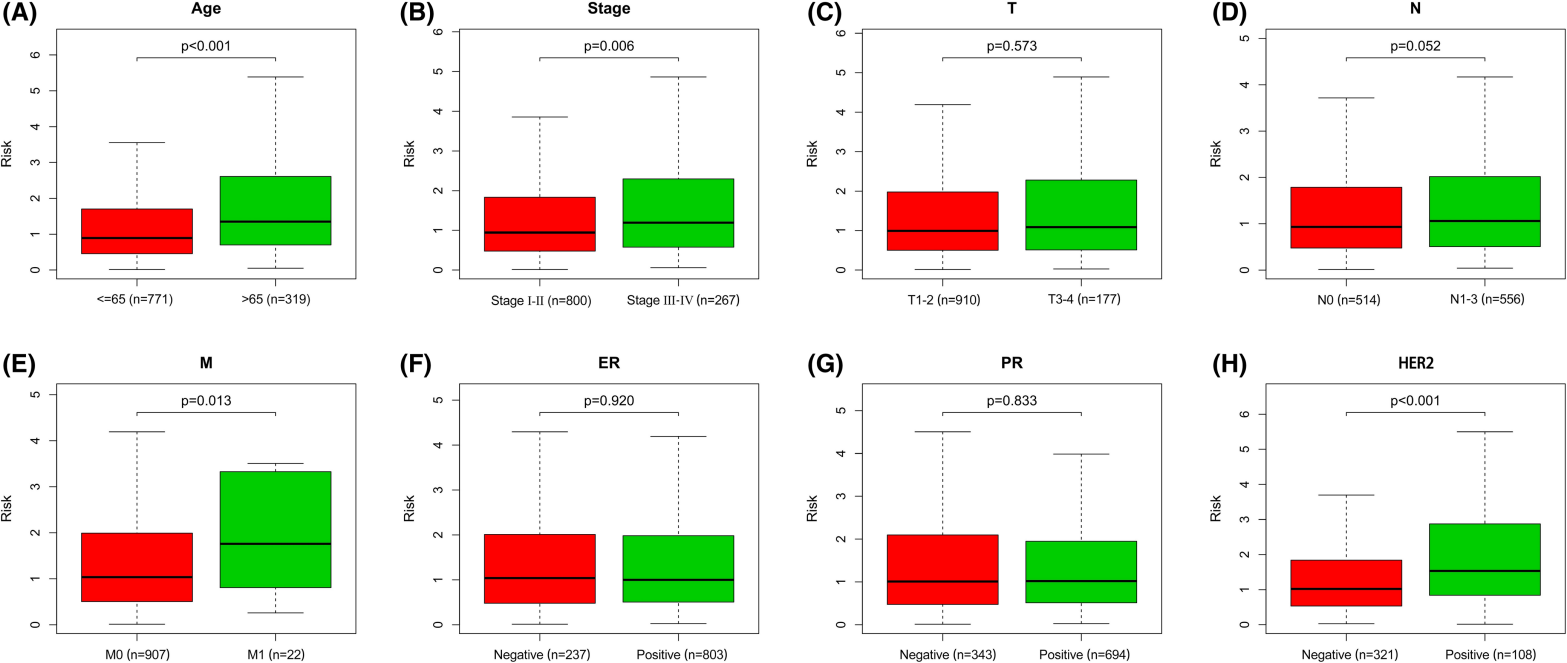
Correlation between risk score and clinicopathological factors. (A) Age. (B) Pathological stage. (C) T stage. (D) N stage. (E) M stage. (F) ER status. (G) PR status. (H) HER2 status

A higher risk score was notably connected to an age > 65 years old, AJCC stage III–IV, distant metastasis, and positive HER2 status (Figure 3A,B,E,H). There was, however, no link between risk score and T and N stage, as well as with ER and PR receptor status (Figure 3C,D,F,G).

### 3.6 The predictive reliability of the 16‐AHGsignature

**FIGURE 4:**
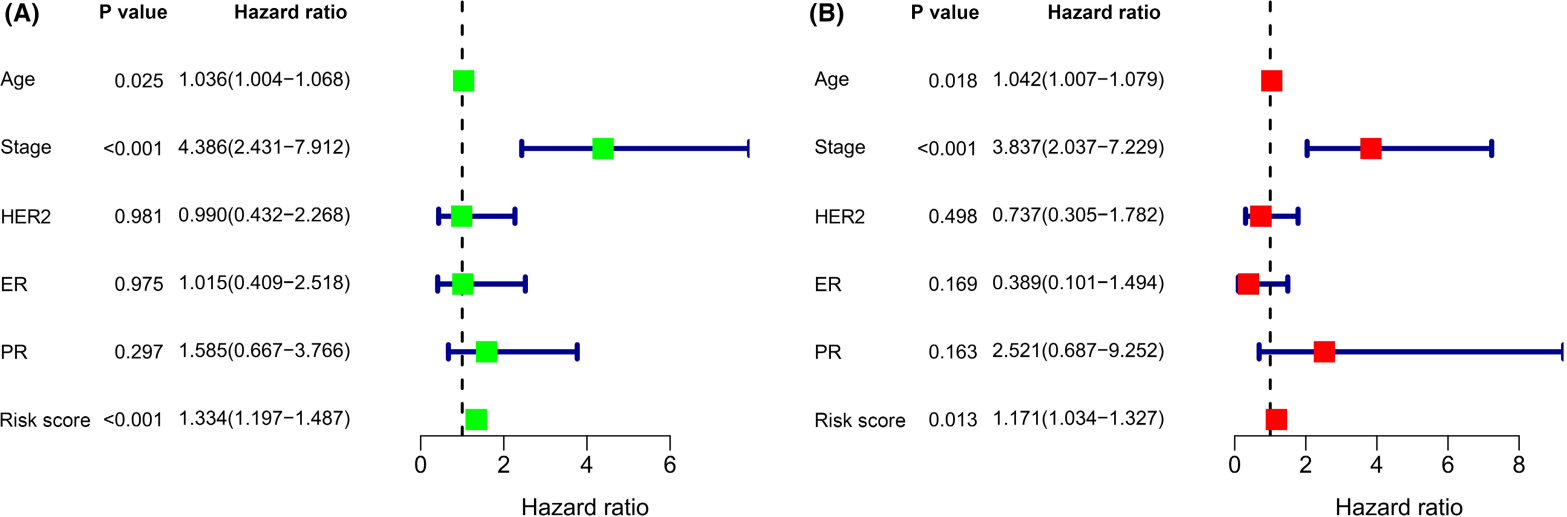
The 16‐AHG signature is an independent prognostic factor for BC patients. (A) Univariate Cox regression analysis. (B) Multivariate Cox regression analysis

**FIGURE 5:**
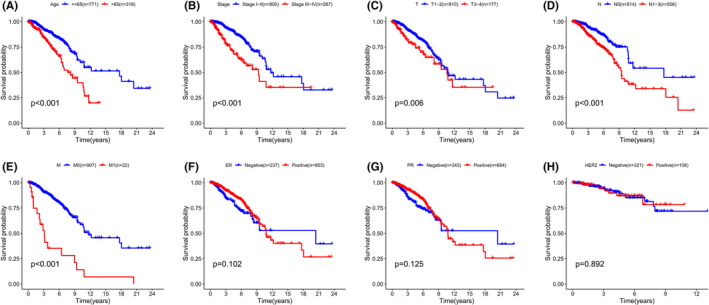
Kaplan–Meier survival analysis for predicting survival in BC patients with different clinical features. (A) Age. (B) Pathological stage. (C) T stage. (D) N stage. (E) M stage. (F) ER status. (G) PR status. (H) HER2 status

**FIGURE 6:**
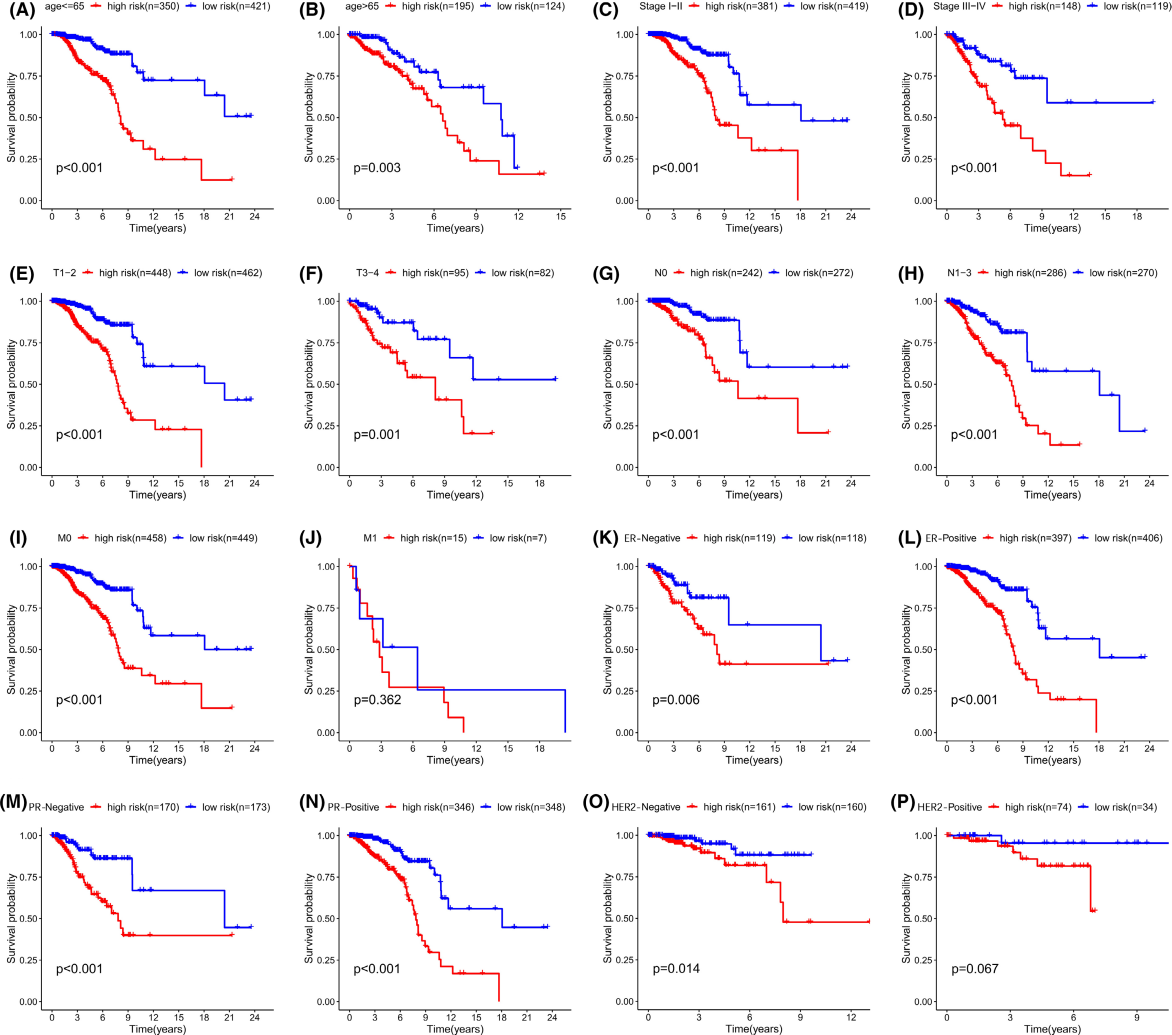
Kaplan–Meier subgroup analysis based on the 16‐AHG signature in BC patients stratified by clinical characteristics. (A) Age < =65y. (B) Age > 65y. (C) Early stage (Stage I‐II). (D) Advanced stage (Stage III‐IV). (E) T1‐2. (F) T3‐4. (G) N0. (H) N1‐3. (I) Patients without distant metastasis. (J) patients with distant metastasis metastasis. (K) ER‐Negative. (L) ER‐Positive. (M) PR‐Negative. (N) PR‐Positive. (O) HER2‐Negative. (P) HER2‐Positive

To determine whether the 16‐AHG signature could predict the outcome of BC patients independent of clinicopathological features, we performed univariate and multivariate regression analyses with gene signature, age, stage, and the status of the ER, PR, and HER2 receptors as covariates. Age (HR = 1.036, 95% CI: 1.004–1.068, *p* = 0.025), stage (HR = 4.386, 95% CI: 2.431–7.912, *p* < 0.001), and risk score (HR = 1.334, 95% CI: 1.197–1.487, *p* < 0.001) were all found to be associated to BC OS in univariate regression analysis (Figure 4A). Multivariate analysis showed that age (HR = 1.042, 95% CI: 1.007–1.079, *p* = 0.018), stage (HR = 3.837, 95% CI: 2.037–7.229, *p* < 0.001), and risk score (HR = 1.171, 95% CI: 1.034–1.327, *p* < 0.001) were independent prognostic variables for BC patients (Figure 4B). Patients over 65 years old, AJCC stage III–IV, T3–4, lymph node metastasis, and distant metastases had a worse outcome, according to the KM survival curves (Figure 5A–E). Meanwhile, there were no significant correlations between the ER, PR, and HER2 receptor status and the prognosis of BC patients (Figure 5F–H). We then conducted separate analyses to test the predictive ability of the gene signature in subgroups with different clinical characteristics. In age, T stage, N stage, ER status, and PR status stratification, lower risk scores were associated with improved survival rates (Figure 6A–H, K–N). However, the gene signature played different roles in distant metastasis and HER2 status. In patients without distant metastasis, the low‐risk group had better OS (Figure 6I), while in patients with distant metastasis, there was no difference in the OS between the two risk groups (Figure 6J). In HER2‐negative patients, the higher risk was significantly associated with worse OS (Figure 6O), while there was no substantial variation in the OS between the different risk categories in the HER2‐positive subgroup (Figure 6P).

### 3.7 Immune infiltration differences between high‐ and low‐risk groups based on the 16‐AHGsignature

**FIGURE 7:**
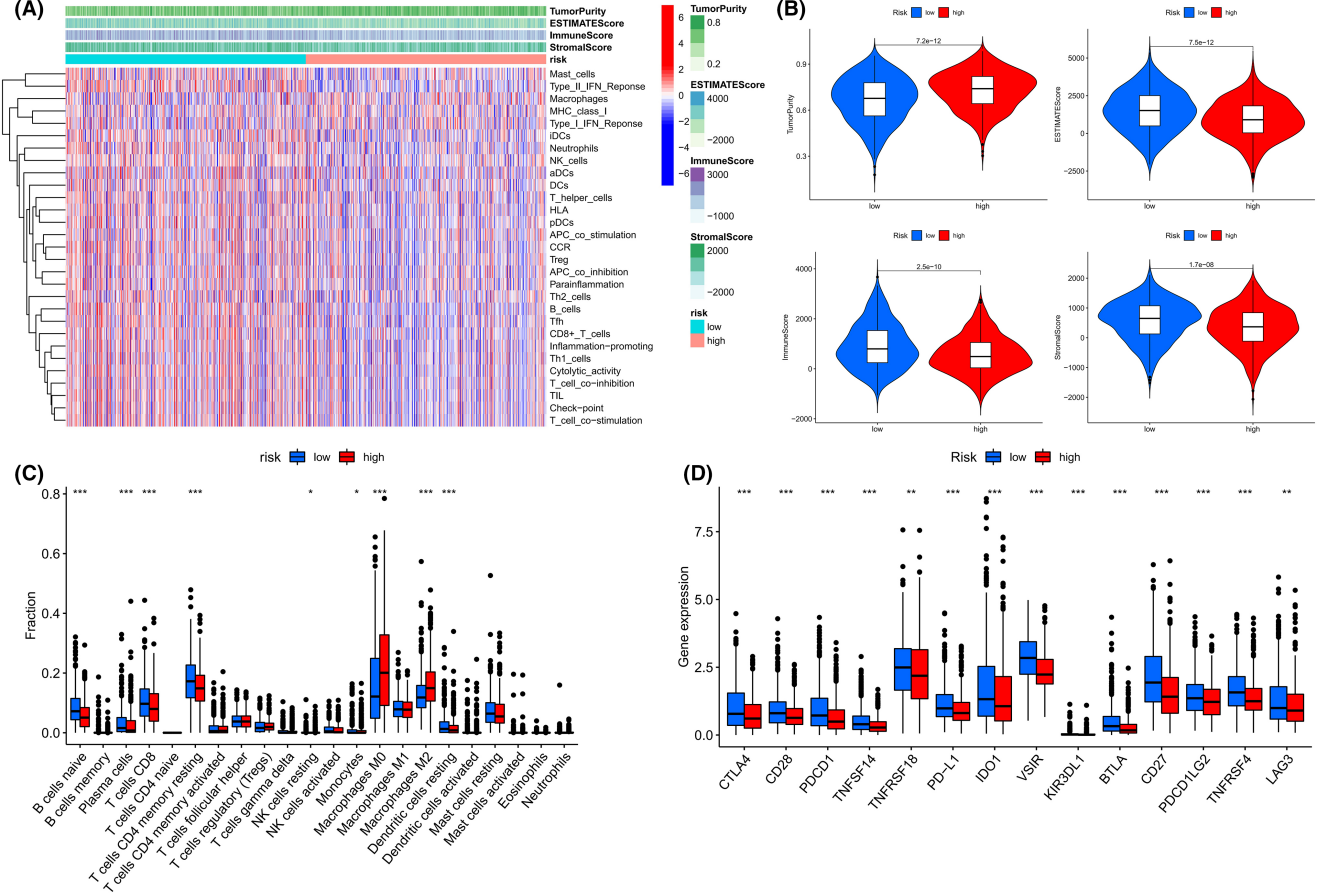
The difference in immune infiltration at high‐ and low‐risk groups based on the 16‐AHG signature. (A) The infiltration of 22 immune cell subtypes and seven immune‐related pathways in high‐ and low‐risk groups was analyzed by ssGSEA. (B) The relationship between risk score and tumor purity, immune score, stromal score, and corresponding estimated score. (C) Difference in infiltration fractions of 22 immune cell subsets in high‐ and low‐risk groups. (D) The expression levels of 14 immune checkpoint genes in different risk subgroups. (*p < 0.05, **p < 0.01, and ***p < 0.001)

**FIGURE 8:**
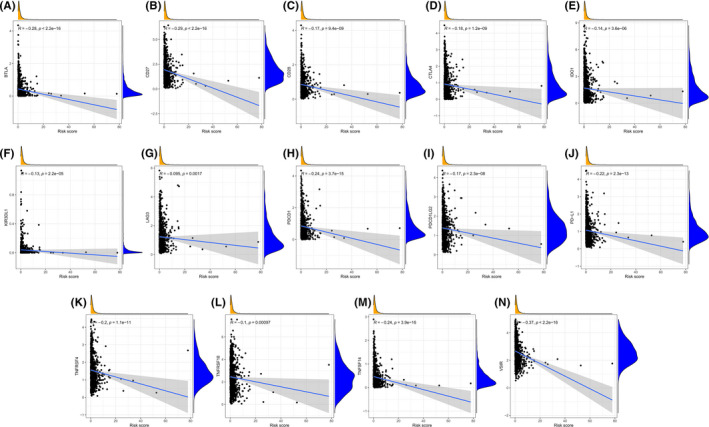
Correlation between expression of 14 immune checkpoints and risk score based on the 16‐AHG signature. (A) BTLA. (B) CD27. (C) CD28. (D) CTLA4. (E) IDO1. (F) KIR3DL1. (G) LAG3. (H) PDCD1. (I) PDCD1LG2. (J) PD‐L1. (K) TNFRSF4. (L) TNFRSF18. (M) TNFSF14. (N) VSIR

The infiltration of 22 immune cell categories and seven immune‐related pathways in all BC patients was investigated using the ssGSEA technique. In low‐risk individuals, the heatmap revealed high levels of immune infiltration (Figure 7A). Risk score was shown to be inversely connected with stromal, immune, and corresponding estimate scores, but favorably correlated with tumor purity using the “estimate” algorithm and unsupervised consensus cluster analysis (Figure 7B). The “CIBERSORT” algorithm was applied to estimate the infiltration difference among the 22 immune cell subsets in these two risk groups. The high‐risk group had a larger proportion of M0 (non‐polarized) and M2 macrophage infiltration and a lower fraction of monocytes, naïve B, plasma, resting CD4 memory T, CD8 T, resting NK, and resting dendritic cells (Figure 7C). Moreover, we found that the immune checkpoints expression differed significantly between the two risk groups (Figure 7D). The expression of 14 immune checkpoints (BTLA, CD27, CD28, CTLA4, IDO1, KIR3DL1, LAG3, PDCD1, PDCD1LG2, PD‐L1, TNFRSF4, TNFRSF18, TNFSF14, and VSIR) was notably higher in the low‐risk group, suggesting that they had a stronger immune phenotype (Figure 8). The 16‐AHG signature was shown to identify low‐risk patients who might be candidates for immune checkpoint inhibitors (ICIs).

### 3.8 Establishment of a predictive nomogram model based on the combined apoptosis and hypoxia gene signature

**FIGURE 9:**
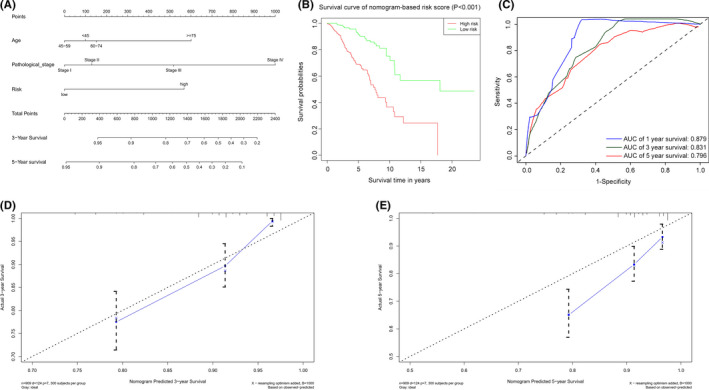
Establishment and verification of a predictive nomogram model based on the 16‐AHG signature. (A) The sum of the scores of each item on the nomogram predicted the probability of survival in 3 and 5 years. (B) Kaplan–Meier survival analysis of BC patients in high‐ and low‐risk groups based on nomogram. (C) AUC of 1‐, 3‐, and 5‐year predictive power of nomogram. (D) The calibration curve of nomogram for predicting 3‐year survival. (E) The calibration curve of nomogram for predicting 5‐year survival

The nomogram model was developed using independent prognostic markers (gene signature, age, and stage) resulting from univariate and multivariate regression studies for quantitative prediction of the 1‐, 3‐, and 5‐year survival rates of BC patients (Figure 9A). Patients were separated into high‐ and low‐risk categories depending on the nomogram's median score. Patients in the high‐risk group had a worse OS than those from the low‐risk group (Figure 9B *p* < 0.001). The AUC of the nomogram's prediction accuracy was 0.879, 0.831, and 0.796 for 1‐, 3‐, and 5‐year OS, correspondingly (Figure 9C). The value of the C‐index was 0.790. The nomogram performed similarly to the ideal model in 3‐ and 5‐year calibration diagrams (Figure 9D,E).

### 3.9 Functional enrichment analysis of DEGsbetween high‐ and low‐risk groups

**FIGURE 10:**
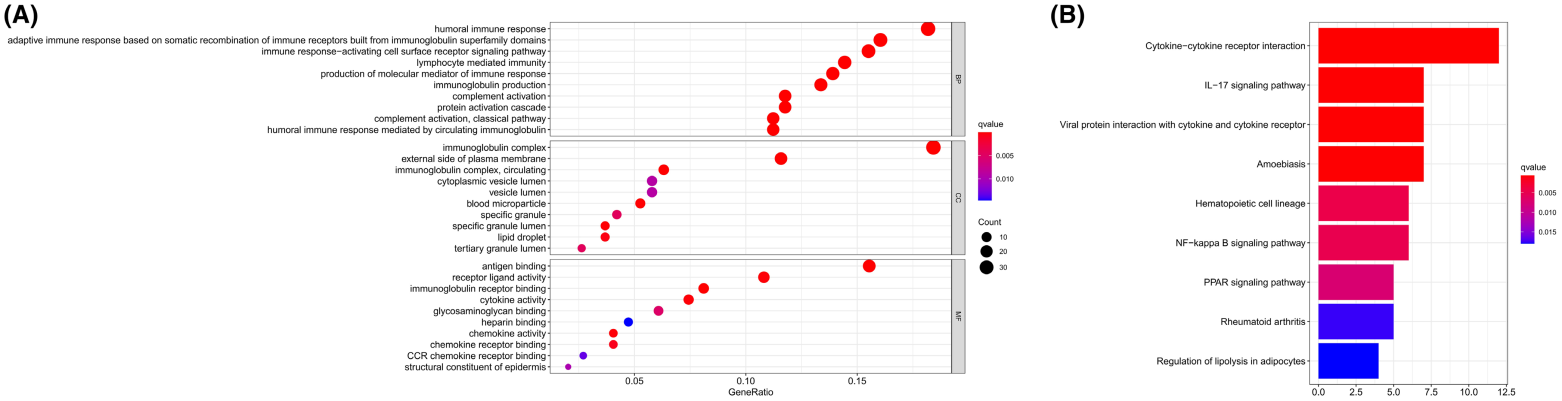
Representative results of GO (A) and KEGG analysis (B)

To explore the relevant biological functions and pathways of different risk groups based on the gene model, GO and KEGG pathway enrichment analyses of DEGs in high‐ and low‐risk groups were performed. The results showed that 217 DEGs were identified between the high‐ group and low‐risk groups, of which 193 genes were upregulated in the low‐risk group and 24 genes were upregulated in the high‐risk group (Table S3). GO enrichment analysis showed that DEGs were significantly enriched in immune‐related molecular functions and pathways, such as humoral immune response, adaptive immune response based on somatic recombination of immune receptors built from immunoglobulin superfamily domains, immune response activating cell surface receptor signaling pathway, lymphocyte‐ mediated immunity, production of molecular mediator of immune response, etc. (Figure 10A). Besides, KEGG enrichment analysis showed that DEGs were also closely related to some immune‐related signaling pathways, such as cytokine−cytokine receptor interaction, IL − 17 signaling pathway, etc. (Figure 10B).

## 4 DISCUSSION

As a complex heterogeneous tumor, BC is underpinned by several molecular mechanisms which have not been clarified. Thus, there are limitations to its early diagnosis and treatment. Clinicopathological features including pathological stage and ER, PR, and HER2 receptor status are now crucial in the diagnosis and prognostication of BC but are insufficient for effective clinical management. Nowadays, with the support of high‐throughput sequencing technology and bioinformatics, several molecular markers have been developed. These have been applied in clinical trials and practices of molecular diagnosis to individualize treatment and predict BC survival.^31^, ^32^, ^33^, ^34^ For example, the 21‐gene recurrence scoring method (Oncotype DX, Genomic Health) is used to develop personalized treatment plans for BC patients who are ER or PR‐positive, HER2‐negative, and lymph node negative by assessing the possibility of recurrence, the potential benefits of chemotherapy, and whether hormone therapy alone can be effective.^35^, ^36^ However, previous studies did not identify apoptosis‐related gene markers. They often analyzed a single gene set, ignoring the important role of hypoxia and the immunologic microenvironment in tumor gene expression. Under severe or prolonged hypoxia, some cancer cells may adapt to escape apoptosis and necrosis, thereby promoting their uncontrolled proliferation.^37^ These anti‐hypoxia‐induced apoptosis cells may have stronger invasive phenotypes and poorer responses to anticancer treatments.^38^ At present, most anticancer therapies, including chemotherapy, radiotherapy, and immunotherapy, work primarily through stimulating cell death pathways.^39^ Recent studies have shown that new anticancer drugs targeting apoptosis pathways have roles in treating cancers of the breast, lung, pancreas, colon and rectum, prostate, head and neck, and blood.^40^, ^41^, ^42^, ^43^, ^44^, ^45^, ^46^, ^47^ Nowadays, the influence of apoptosis and hypoxia on the prognosis of BC is still unclear. Combined analysis of the relationship between AHGs and BC prognosis can better illustrate the effect of regulating hypoxia on cell apoptosis in solid tumors and provide more specific methods for treating solid tumors.

This research examined the predictive power of AHGs for OS in BC patients. We applied univariate Cox regression, LASSO, and multivariate Cox regression analyses to obtain a 16‐gene risk model significantly linked to prognosis (AGPAT1, BTBD6, EIF4EBP1, ERRFI1, FAM114A1, GRIP1, IRF2, JAK1, MAP2K6, MCTS1, NFKBIA, NFKBIZ, NUP43, PGK1, RCL1, and SGCE). According to the survival study, the high‐risk group's OS was considerably shorter. The validation of the ROC curves and ICGC cohort confirmed that the risk model had an excellent forecasting effect on BC prognosis. Clinically, BC is often divided into different subtypes depending on the expression of ER, PR, and HER2 receptors; they are important indicators for the selection of treatment methods, evaluation of malignancy, and prediction of prognosis.^48^ In our study, the 16‐AHG signature, age, and pathological stage can independently predict the prognosis. In addition, through subgroup survival analysis of clinical factors, the risk model was found to be effective in assessing the survival of all other clinical subgroups (age, pathological stage, stage of T, N, status of ER, and PR), except for the HER2‐positive and distant metastatic subgroup. This conclusion still needs to be verified by larger queries, and the possible mechanisms behind it need to be explored. Moreover, the validation of the C‐index, ROC curves, and calibration curves revealed that the nomogram constructed with independent prognostic factors may more accurately and quantitatively predict the future of BC. The AUC of the nomogram in estimating OS in 1, 3, and 5 years was greater than that of the gene signature, indicating that the combination of clinical features was more effective in predicting OS than the gene signature alone. These findings suggested that the 16‐gene risk model is useful not just for prognosis but also for developing personalized therapy approaches for BC patients.

As for the impact of the risk gene model on immune infiltration, the results showed high tumor purity and low immune scores in the high‐risk group. This was opposite to that of the low‐risk group, demonstrating that the immunological state of the two risk groups varies markedly. Fractions of M0 and M2 macrophages in the high‐risk group were significantly upregulated, a finding related to poor prognosis. Macrophages stimulate tumor growth by promoting angiogenesis and chemotherapy resistance in tumor cells, as well as by inducing immune dysfunction through interacting with other immune cells in the TME, resulting in tumor cell immune escape.^49^, ^50^ Given the key functions of macrophages in supporting tumor development in TME, they have become promising targets for immunotherapy.^51^ In contrast, the infiltration of B naive cells, plasma cells, CD4 memory resting T cells, CD8 T cells, resting NK cells, resting dendritic cells, and monocytes in the high‐risk group was considerably lower than that in the low‐risk group, implying that the high‐risk group had immune deficiency. It is reported that in many different types of tumors, strong lymphocyte infiltration indicates good clinical outcomes; this is seen in melanomas, as well as cancers of the head and neck, breast, bladder, urothelium, and ovary.^52^, ^53^, ^54^, ^55^, ^56^, ^57^ Therefore, we infer that insufficient immune infiltration can lead to a poor prognosis. Considering the difference in the fractions of immune cell infiltration compared high‐ and low‐risk populations, these findings are expected to improve the accuracy of immunotherapy with immune cells as the target.

The immune checkpoint pathway has immunosuppressive functions and is involved in immune evasion and progression of tumor cells.^58^ ICIs can relieve this inhibitory effect, activate the antitumor immune response, and eliminate tumor cells. As such, these have gradually become the first‐line treatment for a variety of cancers.^59^ However, certain tumors often respond poorly to ICIs due to factors such as insufficient lymphocyte infiltration in the TME, tumor heterogeneity, and hypoxia‐induced T cell apoptosis; therefore, only some patients can benefit from this therapy.^60^, ^61^, ^62^ Our study found that 14 immune checkpoints were strongly expressed in low‐risk patients, revealing that low‐risk patients will be more responsive to ICIs than high‐risk patients. Previous studies suggested that tumors with abundant immune infiltration had better response to ICIs and better prognosis.^63^ In addition, evidence suggested that high levels of infiltrating lymphocytes in BC patients were associated with PD‐L1 expression and better prognosis.^64^, ^65^ Some clinical trials have shown that the higher the positive rate of PD‐L1, the better the clinical benefit and OS of patients with metastatic TNBC treated with ICI alone or combined chemotherapy.^66^, ^67^ Consistently, GO pathway enrichment analysis reveled that DEGs between different risk groups were obviously enriched in a series of immune‐related biological processes and pathways, indicating that our risk model was closely related to tumor immunity. Some studies suggested that tumor cells may participate in immune escape by resisting apoptosis.^19^ Apoptosis resistance may not only be related to tumorigenesis and chemotherapy resistance, but also affect immune monitoring and immunotherapy. Hypoxia regulates tumor cell metabolism and inhibits tumor cell apoptosis, which not only promotes angiogenesis, tumor cell invasion, and metastasis, but also affects the activation and response of the immune system, drives tumor immune escape, and leads to drug resistance of patients to immunotherapy.^9^, ^20^ KEGG pathway enrichment analysis showed that DEGs in high‐ and low‐risk groups were also significantly enriched in immune‐related signaling pathways, such as cytokine‐cytokine receptor interaction. Twelve DEGs were enriched in this pathway, including CCL17/ CCL19/ CCL21/ CXCL1/ CXCL2/ CXCL13/ IL6/ IL7R/ IL33/ NGFR/ LEP/ LTB. The main components of the set of genes contain many chemokines, such as CC chemokines and CXC chemokines. Chemokines are small molecule secretory peptide, which bind to the G protein‐coupled receptors expressed on the cell surface to induce the targeted aggregation and movement of chemotactic immune cells, thus participating in the immune response, inflammatory response, tumor formation and metastasis, and other physiological and pathological processes.^68^, ^69^ The role of chemokines in tumor immunotherapy has received extensive attention. Many studies have used their chemotaxis characteristics to improve the efficacy of tumor immunotherapy.^70^, ^71^ For example, recent study has shown that anti‐PD‐1 immunotherapy can enhance the efficacy of the adoptive cell transfer therapy by increasing the expression of CXCL10 in tumors.^70^ Totally, immunotherapy guided by the 16‐AHG signature in our study is expected to become a promising antitumor treatment.

The 16‐AHG signature contained AGPAT1, BTBD6, EIF4EBP1, ERRFI1, FAM114A1, GRIP1, IRF2, JAK1, MAP2K6, MCTS1, NFKBIA, NFKBIZ, NUP43, PGK1, RCL1, and SGCE, and the relationship between some genes and BC has been explored in previous studies. Eukaryotic translation initiation factor 4E‐binding protein 1 (EIF4EBP1), as an inhibitor of EIF4E, synergistically induces the expression of c‐MYC and Cyclin D1 with eukaryotic elongation factor‐2 kinase (eEF2K) inhibitor to suppress the growth of TNBC cells.^72^ ErbB receptor feedback inhibitor 1 (ERRFI1) is a negative regulator of cell proliferation proteins. The loss of ERFFI1 expression may be related to the development of invasive BC.^73^ Interferon regulatory factor 2 (IRF2) has been considered a potential tumor protein, which may alter the IFN‐γ/Jak/Stat/IRF pathway through endogenous IFN‐γ, allowing cells to escape growth control mechanisms and promoting stronger invasiveness and faster tumor growth.^74^ Multiple copies of t‐cell malignancy 1 (MCTS1) encode a ribosomal binding protein which regulates the ribosomal cycle, translation restart, and tissue growth.^75^ It was found that the overexpression of MCTS1 in invasive TNBC cells predicted poor prognosis and promoted progression of tumors.^76^ Nuclear factor κ‐β inhibitor α (NFKBIA) regulates the expression of genes involved in cell multiplication, transdifferentiation, apoptosis, and metastasis, and its variation may affect the development of tumors.^77^ However, few studies on this gene polymorphism and BC risk have not observed a significant relationship.^78^ Nuclear pore 43 (NUP43) encodes the Nup107‐160 complex, which is located at the centromere in mitosis and regulates mitosis and chromosome separation.^79^ In luminal A and HER2‐positive BC, the overexpression of NUP43 was significantly associated with poorer OS.^80^ Phosphoglycerate kinase 1 (PGK1) controls ATP production by limiting glycolysis.^81^ PGK1 is a risk gene for the survival of BC, and forms a positive feedforward loop with HIF‐1α to stimulate the progression and metastasis of BC.^82^ A member of the ε subtype of the sarcoglycan family, SGCE has recently been discovered to be overexpressed in BC stem cells (BCSC). It plays important roles in self‐renewal, tumorigenesis and metastasis, chemotherapy resistance, extracellular matrix deposition, and remodeling of BCSC, and was thus associated with poor prognosis.^83^ However, there are few studies on the mechanism of action of AGPAT1, BTBD6, FAM114A1, GRIP1, JAK1, MAP2K6, NFKBIZ, and RCL1 in BC, which is worthy of further study.

Our current research has several limitations. First, the 16‐AHG signature based on the TCGA database was only verified in the ICGC cohort, and it requires large‐scale multicenter prospective queries for further verification. Second, further basic tests are necessary to ensure the bioinformatics results and explore the internal mechanisms of the gene signature. This also provides ideas for us to continue to study this project.

In conclusion, we identified an effective and accurate signature of AHGs in BC. This risk model can not only be utilized to predict prognosis in BC patients and improve clinical management, but also to evaluate the immune microenvironment and identify appropriate patients for immunotherapy.

## CONFLICT OF INTEREST

The authors declare that they have no conflict of interest.

## AUTHOR CONTRIBUTIONS

SL and HK put forward the main principle and guided the research design. XR, HC, JW, and RZ retrieved and analyzed original data. NW, DL, XX, and HZ interpreted the results. XR wrote the first edition of the paper and then DL, XM, and CD modified it. The findings were discussed among all authors, and the manuscript was revised accordingly. The submission of final paper was reviewed and approved by all contributors.

## Supporting information


Figure S1

Figure S2

Figure S3

Table S1

Table S2

Table S3

## Data Availability

This study analyzed publicly available data sets. These data can be found here: https://portal.gdc.cancer.gov and https://dcc.icgc.org/projects/BRCA‐US.
