# Supplementary figures and images for: Identification of a combined apoptosis and hypoxia gene signature for predicting prognosis and immune infiltration in breast cancer
Source: Cancer Med. 2022 Apr 20;11(20):3886–901. doi: 10.1002/cam4.4755 (PMC9582692; doi:10.1002/cam4.4755)

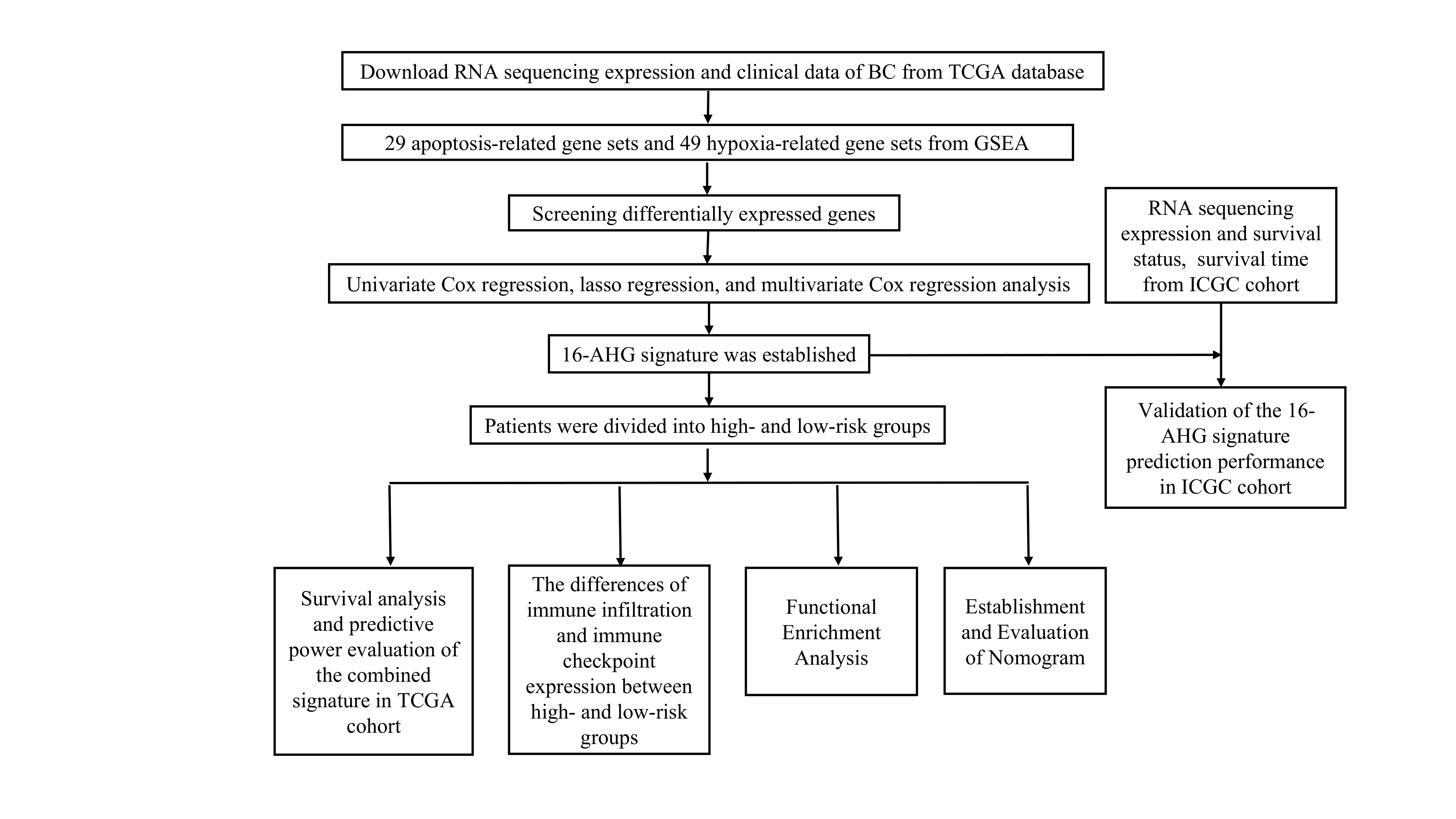

Supplement: Supplementary file 1 — Figure S1 [file CAM4-11-3886-s005.jpg]

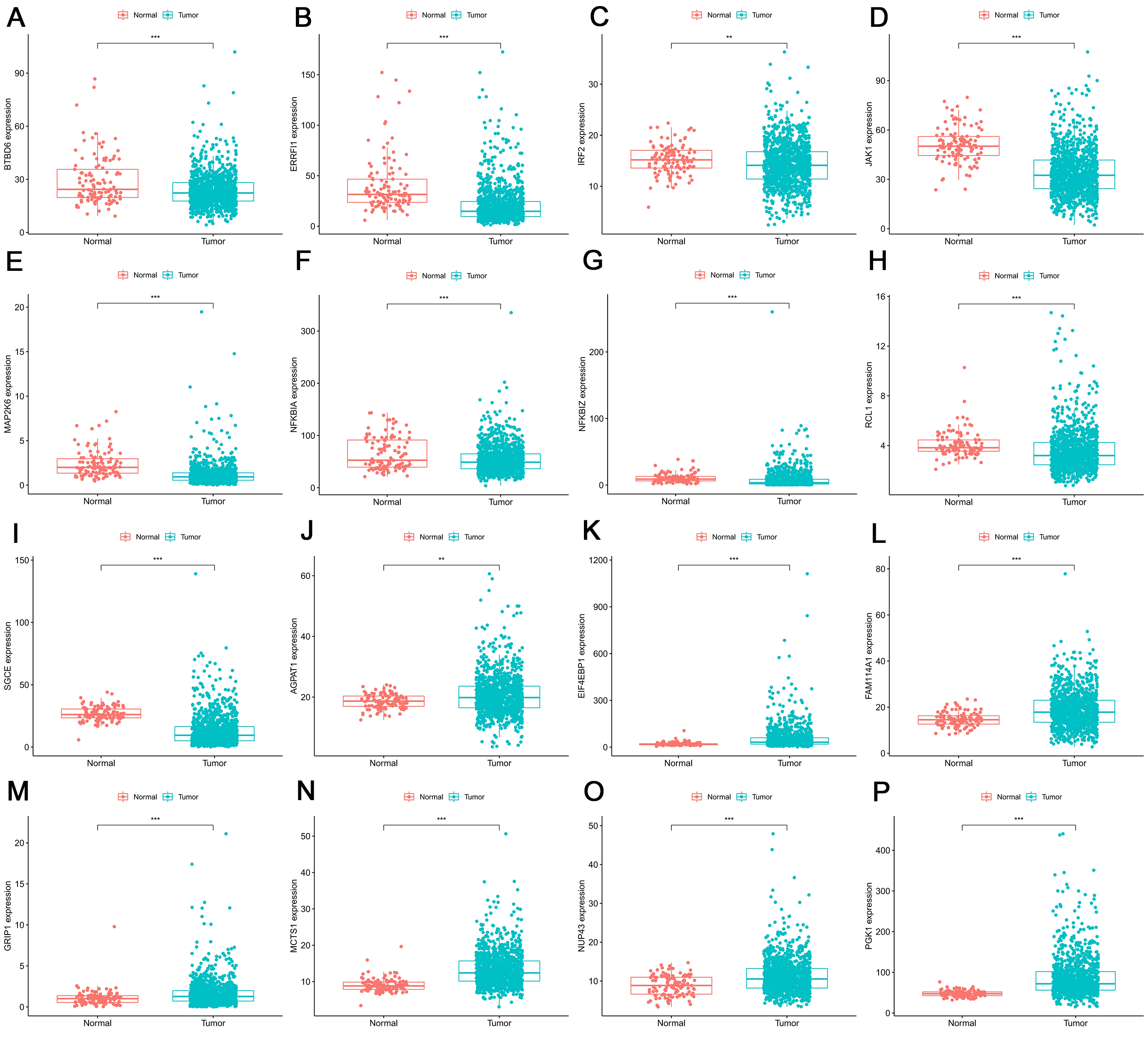

Supplement: Supplementary file 2 — Figure S2 [file CAM4-11-3886-s003.jpg]

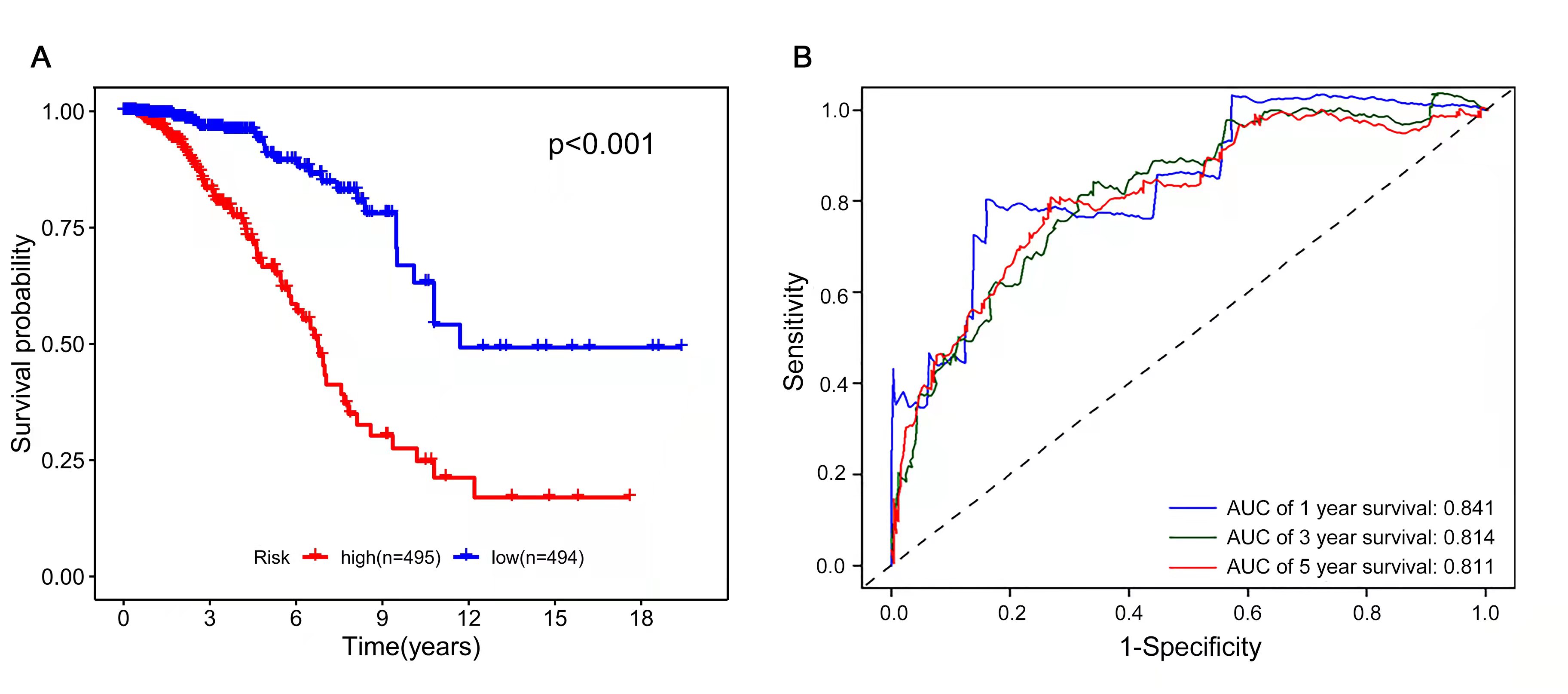

Supplement: Supplementary file 3 — Figure S3 [file CAM4-11-3886-s001.jpg]
