# Supplementary material for: Identification of a combined apoptosis and hypoxia gene signature for predicting prognosis and immune infiltration in breast cancer
Source: Cancer Med. 2022 Apr 20;11(20):3886–901. doi: 10.1002/cam4.4755 (PMC9582692; doi:10.1002/cam4.4755)
Supplement: Supplementary file 4 — Table S1 [file CAM4-11-3886-s004.doc]

**Supplementary table 1.** Differentially expressed apoptosis and hypoxia-related genes between BC and normal samples.

| **Down-regulation （N=1805）** | | | | | | | | | |
| --- | --- | --- | --- | --- | --- | --- | --- | --- | --- |
| LEP | PCK1 | RBP4 | FHL1 | LPL | LDB3 | LIPE | CHRDL1 | ATP1A2 | ITIH5 |
| SCGB1A1 | DES | AOC3 | CSF3 | HIF3A | LRRC3B | AMPD1 | GPX3 | SORBS1 | CDO1 |
| ACACB | ITGA7 | NPR1 | MASP1 | PGM5-AS1 | CXCL2 | PDK4 | AKR1C1 | DPT | PFKFB1 |
| SAA1 | MME | CAV1 | PPARG | TSLP | MYH11 | MAOA | SYNM | TMEM100 | MYZAP |
| KIT | PENK | ADAMTS5 | GDF10 | TRIM63 | SGK2 | TNS1 | ZBTB16 | KBTBD13 | DMD |
| CFD | ANGPTL1 | LEPR | SAA2 | CARMN | HLF | SYNE3 | CAV2 | ALDH1A2 | KCNB1 |
| IL6 | CRYAB | FEZF2 | KCNA2 | IL33 | ADAM33 | MAB21L1 | ENPP2 | TP63 | BMX |
| SFRP1 | LMOD1 | ANGPT1 | EGR1 | FGF2 | GPR146 | GIPC2 | AKAP12 | LINC00844 | GPLD1 |
| DDR2 | MYOM2 | FHL5 | PDZD2 | KCNMB1 | KLHL29 | KLF4 | ECM2 | FOS | CX3CL1 |
| SRPX | GFAP | PGM5 | ALDH1A1 | AK5 | GSN | SPRY2 | DST | TGFBR3 | SLC29A4 |
| HOXA5 | MFAP4 | GNAI1 | FLNC | FAM13A | IGF1 | SYNPO2 | EPHB1 | ANXA1 | HOXA4 |
| CLMP | RYR3 | FAT2 | GPR182 | NRG1 | NSG1 | AKR1C3 | MYLK | C2orf88 | MYOCD |
| NDRG2 | GABRE | PROX1 | FAM189A2 | ITM2A | PREX2 | ANK2 | PDLIM3 | NRN1 | SCN9A |
| AOX1 | NR3C2 | TGFBR2 | EGR3 | MAGI2-AS3 | DEPP1 | MRAS | ZFP36 | DENND2A | ALDH2 |
| ALPK3 | CACHD1 | MGLL | TF | GNG11 | KCNH8 | ATF3 | ABCB1 | C6orf58 | FAM162B |
| MCAM | CETP | CEBPA | GNAL | GPRASP1 | PPP1R12B | LRRN3 | CREB5 | WIF1 | CDKN1C |
| JCHAIN | NOS1 | LHFPL6 | STX11 | VWF | MRGPRF | CXCL3 | AKAP6 | ACTG2 | CFL2 |
| S1PR1 | TXNIP | PRELP | DUSP1 | SPTBN1 | ID4 | PTGIS | CXCR2 | PLPP3 | FFAR4 |
| MAPK10 | KRT14 | CXCL12 | KRT5 | EFEMP1 | CSRNP1 | EPAS1 | ALDOC | NR4A1 | PKDCC |
| PDGFD | EHD2 | GYPC | MMRN2 | ANGPTL4 | SOSTDC1 | ROBO4 | FERMT2 | HTR4 | PFKFB3 |
| CAVIN1 | SOCS3 | ACSM3 | CELF2 | TM4SF20 | HSPA12A | GPM6B | PTPRB | TRIM29 | RCAN1 |
| KANK1 | SYNPO | TEK | PRIMA1 | FBLN5 | ANGPTL2 | KANK3 | TRPM6 | SPRY1 | ALDH1A3 |
| CCDC80 | CTSG | MSRB3 | HAP1 | TACC1 | ADCY4 | PELI2 | METTL7A | ACKR3 | TNFRSF10D |
| TNS4 | AIFM2 | CEP112 | F3 | CNRIP1 | KRT17 | PCDH9 | PLEKHH2 | NECTIN3 | STBD1 |
| CRIM1 | PLAGL1 | IRX1 | LAMB3 | ITSN1 | F8 | CLIP4 | ACTA2 | NFIB | VLDLR |
| MIR100HG | NR3C1 | ZNF204P | RAPGEF3 | KRT15 | GRK3 | PTGS2 | CD248 | MT1X | PTPN14 |
| C7 | SSPN | CD302 | LARP6 | NEXN | RHOJ | ACSS2 | C2CD2 | COL14A1 | EPB41L2 |
| AGPAT2 | SAMD4A | APOD | LRP1 | MEIS2 | SEMA6D | SOBP | GRAMD2B | TAC1 | MEST |
| VSIR | SEMA5A | C1QTNF1 | MIR22HG | TCF15 | FOXN3 | CRHR2 | RIMS3 | YPEL4 | NPR2 |
| EGF | CUBN | NCAM1 | RGCC | IGFBP1 | ME1 | DPP4 | MEG3 | TMEM35A | ARRDC3 |
| TREH | DUSP6 | SGK1 | MAFF | GAS2 | NOTCH4 | ZCCHC24 | AHNAK | THRB | GABARAPL1 |
| STOX2 | PKD2 | ZEB2 | AMOTL2 | SELE | PIK3R1 | MET | DNAJB4 | FLG-AS1 | TMEM71 |
| SCD | PLA2G4A | NDNF | RND3 | PRKD1 | MID1 | EGFR | LAMA4 | TTC28 | ABLIM3 |
| DIXDC1 | BACH2 | FHOD3 | ALOX15B | JUN | PTGER4 | ANO6 | PTCH1 | ADGRA2 | SCGB3A1 |
| LAMC2 | IRS2 | GBE1 | ICAM2 | ANKDD1A | ADD3 | FEZ1 | EDN1 | MYL9 | GULP1 |
| CSRP1 | ADM | PDGFRA | COL4A6 | SYNE1 | RARRES2 | NAV2 | RRAD | TAGLN | DPYSL2 |
| GIMAP7 | CCDC136 | VIM | NEDD9 | ARHGEF6 | KLF6 | TPM2 | EOGT | SCHIP1 | TCEAL2 |
| GIMAP1 | PPARA | KAT2B | RNF125 | ANKRD35 | PDGFA | ARHGAP10 | ANXA3 | CD200 | LRCH2 |
| DKK3 | ADAMTS3 | SEMA6A | FILIP1L | PLTP | VSTM4 | LMO3 | F13A1 | SLC47A2 | CPAMD8 |
| CHST3 | HOXA10 | FZD7 | SOCS2 | PPL | ETS2 | TRPC1 | GPRC5B | KCNIP1 | PPP1R15A |
| CCDC50 | USP53 | NT5E | IL13 | IGHA1 | PDLIM4 | RASA4 | TIPARP | KIRREL1 | PKNOX2 |
| THSD7A | ARHGEF40 | CAB39L | KLF11 | KLF10 | SACS | PECR | GSTM2 | CDKN2C | ITGA9 |
| PHLDB2 | ABCA1 | SERPINF1 | BHLHE41 | CYP39A1 | TBC1D4 | SLC6A2 | MEF2C | UGP2 | GRK5 |
| LMO2 | MECOM | ITGA1 | EPHA2 | PDGFRL | PTX3 | ETV1 | SV2C | QKI | ZFP36L2 |
| STAT5B | GAL3ST1 | AFP | DAB2IP | ACSL4 | C20orf194 | TUBB6 | PAX3 | BOK | ARID5A |
| ADAMTS9 | MMP19 | INPP1 | RRAGD | CDH22 | SIRPA | ID1 | DDIT4L | FLI1 | STXBP1 |
| POU6F1 | PYGL | MAP1B | CDC42EP2 | NEURL3 | KLHL21 | PODN | CH25H | GSPT2 | AVPI1 |
| RASL12 | ECHDC1 | CLCNKB | BCL6 | ARHGAP26 | PGM1 | OLFML2A | LIMCH1 | YAP1 | ACAA2 |
| PXDC1 | PRKCA | CCL2 | S100A4 | KLF8 | FBXO32 | TUBB8P11 | TWIST1 | PRICKLE2 | CTIF |
| THBD | LAMC1 | RBMS1P1 | C3 | KANK2 | KDR | LPAR6 | MAP2K6 | SORBS2 | PLPPR4 |
| MXRA7 | KLHDC1 | RCAN2 | MT1L | PARM1 | SATB1 | MITF | DHRS3 | SGCE | DIAPH2 |
| AK4 | GEM | CRTAP | HBEGF | HGF | TK2 | WWC2-AS2 | RASA3 | LRP4 | MICAL3 |
| PCCA | PTGDS | HOXD10 | PEMT | INHBB | PPM1F | FZD5 | OR7E36P | RRAS2 | MYC |
| FMNL2 | KCNJ8 | ERRFI1 | SAV1 | MAP3K8 | CCND2 | NLRP1 | RIPK4 | CTNNAL1 | SGCB |
| TCN2 | SHMT1 | GABRP | BEND5 | ATP2B4 | ARHGEF10 | ESYT1 | MYADM | ITPR1 | FXYD6 |
| DSC3 | NTN4 | FSCN3 | LPIN1 | MXI1 | IL1R1 | KPNA5 | CD93 | ULK4P3 | YBX3 |
| LCAT | PCDHGC3 | EVA1C | FGD5 | MFNG | ITGA6 | PARVA | PGAP1 | LY75 | FOXE1 |
| MSX1 | SLC14A1 | CFLAR | RPS6KA2 | TFPI2 | EHF | SVIL | NFASC | MAP3K5 | PPARGC1B |
| PER3 | MEIS1 | RAG1 | TMEM140 | LPAR1 | FAT1 | TUB | SLC12A4 | CUTC | NFIL3 |
| HOXD4 | CD99L2 | RBPMS | EPHA5 | LDHB | RAPGEF2 | GRAMD1C | HOXD9 | AK3 | GPR87 |
| PCYT1B | TMEM237 | PLIN2 | RBMS1 | TMEM245 | CCDC9B | NAB1 | MBNL1 | SNAI2 | ASPH |
| AKT3 | SPEG | ACSL5 | BNIP2 | FGF9 | MLYCD | SPATA18 | TGFB1I1 | TGFA | IFNGR1 |
| NR1D2 | CYB5R3 | GJC2 | RAMP2 | TCF7L1 | PTPRG | DAPK2 | FYN | KLF7 | SETD7 |
| BCL2L2 | NEK7 | THRA | PNRC1 | KBTBD11 | CDH4 | JAZF1 | KIAA1671 | PTHLH | WTIP |
| IL6R | NHSL2 | FBXO17 | VEGFB | MERTK | C1R | CCDC68 | LAYN | VWA5A | TRO |
| FKBP5 | LGALS3 | RFTN1 | CLCN4 | UACA | TCF4 | BDH2 | ALDH3A1 | SLC2A3 | STAB1 |
| PDK2 | CHRM1 | TNFSF12 | TSPYL2 | ZBTB47 | C14orf28 | CASP10 | SNRK | ARRB1 | SIDT2 |
| CSGALNACT1 | RPS6KA5 | PRSS12 | PKP1 | TCF7 | ANO1 | CALD1 | GNG12 | SH3GLB1 | PHACTR2 |
| HSPG2 | H6PD | FLT1 | HRH4 | SEC14L1 | ARHGAP23 | ALDH4A1 | KLHL2 | NUAK1 | SPNS2 |
| DSE | RAB11FIP2 | PLCG2 | CDC42EP3 | BCL6B | CSF1 | JMJD1C | MYO1C | CYP3A5 | B3GNT5 |
| SNX33 | LRAT | JCAD | NLRP14 | JUNB | KLF13 | WEE1 | WASF2 | ZNF710-AS1 | PIK3IP1 |
| VEGFC | HEG1 | CXCL6 | SRSF5 | ARL10 | DYNC1I1 | PHLDA1 | TBC1D2B | BNIP3L | TMEM200B |
| ITGB3 | ACAT1 | C1S | CREBL2 | RRAS | ARMCX4 | PRKG2 | TAS2R10 | ITGB1 | NIPAL1 |
| HLA-E | PDGFC | SCPEP1 | MBP | CCNG1 | CLK1 | PAM | SOD2 | TIMP2 | TSPAN18 |
| PINK1 | TNFRSF1B | FRMD6 | MMP24 | FABP5 | ACER2 | ZNF395 | ARL4C | ST3GAL6 | ETS1 |
| PCGF5 | ATP8A1 | TNFRSF21 | OTUD3 | CITED2 | NYNRIN | PDLIM1 | MAP1A | PRKAA1 | GBP2 |
| SLC23A2 | GAS6 | ZFYVE9 | PLLP | HIC1 | NR4A2 | FBN1 | ANPEP | SECISBP2L | PTAR1 |
| DENND5A | TFDP2 | NLRX1 | COL15A1 | TRIOBP | SLC25A20 | ZMAT3 | TLE2 | NOVA1 | DUSP2 |
| VSNL1 | FAM171A1 | RNF38 | ACADVL | SKI | RORA | GDF7 | SEMA3E | SNTA1 | PDPR |
| ARHGAP29 | PODXL | PMP22 | PJA2 | RPL5 | ANKMY2 | VCL | DYRK3 | MAP3K3 | TTLL11 |
| SLC25A16 | TFAP2C | SLCO4A1 | CTDSP2 | CD59 | ZBTB44 | RBL2 | ARHGAP5 | ITGA8 | MYH10 |
| KCNIP4 | SLC16A2 | DENND4C | IL7 | MTUS1 | BAZ2B | RIMKLB | AXL | SCARA3 | CARF |
| DDX3Y | TNIP1 | WWTR1 | CSDE1 | NECTIN1 | STARD13 | FGFR1 | ANXA5 | TSPAN4 | SWAP70 |
| CACNA1C | MYO15B | SYT2 | CYTH3 | GPD1L | SNX1 | OPTN | ENPEP | DCHS1 | WSB1 |
| JAK1 | PPTC7 | AFF1 | NGF | N4BP2L2 | LBH | AKAP11 | ANG | SLFN5 | VPS13D |
| ZNF10 | TNFAIP8 | NISCH | PTPN11 | FAS | FBXL3 | HBP1 | WNT2B | TJP1 | ALDH7A1 |
| LRP5 | ITGB4 | AMOT | KIF1B | CLU | CHST2 | ALDH3A2 | CCNB1IP1 | CPVL | LYRM9 |
| STOX1 | LRP6 | TMX4 | ELMO1 | MT-ND4 | LAMB2 | TFCP2L1 | IGFBP3 | HIVEP2 | TSHZ1 |
| DCC | RUSC2 | CARD8 | LRRC32 | BCKDHB | ITPR2 | KSR1 | C11orf95 | LPCAT4 | SHOX2 |
| MPZL2 | SYNC | AC099548.2 | TSC22D3 | RADIL | SIK1 | PDE8A | OSBPL9 | MAP2 | EPB41L3 |
| PRKCH | SPON1 | EYA4 | BX842568.2 | USP31 | ITGA2 | GPX2 | AGO4 | FERMT1 | EVC |
| NNMT | SOS2 | ENG | DDX3X | RAPGEF1 | IGF2 | MEF2A | SIK3 | NMRK1 | CCSER2 |
| CASP1 | FLNA | HSPA4L | SLCO3A1 | EFNA5 | LHFPL2 | TPM1 | COL7A1 | PRSS16 | DHRS9 |
| SNHG5 | TJP2 | CEBPD | TUBB2A | GPNMB | PRKCQ | ARHGEF7 | PPP1R12A | ADCY3 | GSTA1 |
| CAPN5 | FZD1 | POLR1E | RAVER2 | TSHZ3 | ATM | POLR3GL | HERC3 | EFEMP2 | IL1RAP |
| THYN1 | ATG2B | EEF1AKMT3 | PHLDA3 | ADARB1 | TIMP3 | JUND | EIF1B | NFE2L2 | TRIM22 |
| GNAQ | FAM241A | RPL22 | CCDC107 | HIBCH | TLE1 | CSRP2 | CAST | HOXD8 | MAP4 |
| ADORA2B | ISCU | AGPAT4 | PIM1 | ZNF214 | NACC2 | NFKBIZ | CORO1C | MAPK11 | GPR155 |
| IMMP2L | CPEB3 | PLCD1 | DUSP3 | INPP5D | RPS27A | HEY1 | SLC31A2 | IGF2BP2 | FGF11 |
| SLC6A16 | NID1 | JAG1 | RPL3 | MAP1LC3B | AACS | OSTM1 | FOXO3 | VPS51 | MCFD2 |
| TRAF6 | HEPH | C4orf3 | BTG1 | GJA5 | KDSR | PXN | DISC1 | MAPRE2 | PKD1 |
| POLK | HACL1 | ZNF22 | KCNMA1 | ATG14 | POLR2M | SELENOV | ID3 | DPYSL3 | RBPJ |
| ADD1 | ENDOD1 | PPP3CB | TPST1 | SMIM8 | AKAP1 | MAP7D1 | IER2 | LNPEP | ERICH1 |
| LDLR | ATXN3 | NFIA | IFI16 | PPP2CB | IFRD1 | ZNF189 | ZBTB20 | WHAMM | KCNN3 |
| ECE1 | SMTN | EPS15 | CRK | GPC6 | PLD2 | RUBCNL | SV2A | JAK2 | MKLN1 |
| IFFO1 | MPP1 | NOS3 | GNS | KLHL20 | KLHL24 | PPP3CC | KIF5A | MAP3K14 | PRDM2 |
| CD81 | NHS | SLITRK5 | XPC | CYS1 | APPL2 | SDCBP | STOM | ANGPT2 | GUSBP11 |
| VAMP5 | USP34 | ACOT2 | TTLL3 | FGFRL1 | CASP4 | RYK | STAT4 | RASGRP2 | APC |
| LRIG1 | CBX6 | SH2B3 | ICA1L | ZNF292 | C2orf49 | VWC2 | PAN2 | FABP5P3 | KCNN4 |
| BMT2 | CRYZL1 | BBX | HSPB8 | AKR1B1 | CLDN15 | SMAD6 | SLC25A36 | CLIC4 | GMFG |
| RIPOR2 | DDX19B | TM4SF1 | ZNF655 | SH3BP2 | HDAC5 | MAST4 | FBXO8 | CDKN1A | CD40 |
| ITGA3 | PDE4B | TNFRSF17 | ARL6IP5 | SDC2 | MLXIP | NOTCH1 | NEDD4 | RGS3 | TMEM218 |
| NUDT11 | DPY19L1P1 | BCL2L10 | LYST | RSL24D1 | OSR2 | TOB2 | BCL11A | PHYH | SLC46A3 |
| SETX | CYP3A7 | RHOBTB3 | GLIS1 | SORBS3 | ROCK1 | VWA8 | NEK3 | SLC47A1 | GRHL1 |
| OGT | DOCK7 | CARD10 | IDH1 | CCPG1 | IL16 | MTHFD1 | ST3GAL5 | SRGN | FBXO2 |
| CCDC102A | GPER1 | DUSP7 | CHIC2 | CDS2 | TES | SETMAR | PAQR3 | SMAD5 | ZBTB1 |
| FNDC3B | VPS11 | ACTN1 | AJUBA | GLIPR2 | CTH | SLMAP | SYTL3 | MYOF | ZNF229 |
| B4GAT1 | MCL1 | DAAM1 | NOTCH2 | PTPRVP | RAPGEF5 | BAG5 | RGS13 | PALLD | ZNF330 |
| TIAF1 | DCUN1D4 | SLC29A1 | SARM1 | FTL | ZYX | CREG1 | CEPT1 | TUBGCP6 | CHPT1 |
| YWHAG | GPALPP1 | GCLC | RTN1 | IL18R1 | EEF2K | MAP3K4 | LINC00222 | PTPN13 | SIRT2 |
| PKP2 | LGALS1 | NIPAL3 | TGDS | NEDD4L | HOOK3 | ROBO1 | SPAG9 | RLF | PPP1R3B |
| ATRX | YPEL5 | UBE4B | COPS2 | KLRC4 | RIC1 | BTBD6 | OTUD4 | CTNNB1 | RNF13 |
| CD47 | CCNH | DPH3 | PPP3CA | HOOK2 | IFNAR1 | HNRNPDL | SGSM2 | MAT2B | LGALSL |
| CCND3 | NAMPT | EID1 | CALM1 | FRZB | RHEBP1 | GCLM | PHF1 | RAP1A | DUSP14 |
| MT-CO1 | TAB2 | TAF1 | USP13 | CTDSP1 | ACOT9 | DSG2 | SLC43A3 | COL4A2 | SMURF2 |
| KCNK2 | RBM33 | GPATCH8 | PLEKHA2 | ABI2 | ATP7A | SERTAD1 | NFKBIA | TSPOAP1 | CCRL2 |
| IRX2 | SCAF11 | DDX19A | ITPKB | NHEJ1 | SF3A1 | DEPTOR | SLC25A12 | SORL1 | CXCL1 |
| SOX9 | NMT1 | AHSA2P | DDB2 | RGMB | RALGDS | BCL2L13 | PEX3 | USP47 | DCTN6 |
| SLC12A2 | RAD54L2 | DAPL1 | ACER3 | TNKS | S100A6 | ASXL1 | IREB2 | TOR1AIP1 | SNX9 |
| CNTNAP4 | ZFYVE1 | RNF185 | TRMT5 | GADD45A | GATM | RPH3AL | SUPT3H | BMP4 | WASHC4 |
| RXRA | STK17B | DTNA | LSM6 | MINK1 | ARHGEF17 | PARP4 | EIF2B3 | RCHY1 | IL15 |
| ZNF518A | FOXJ3 | BIN3 | S100A10 | MAP4K5 | SYF2 | SCARB1 | TSGA10 | ANKRD37 | SLC41A2 |
| ABL1 | NEURL1B | SERPINB8 | GSTP1 | FOSL2 | KCTD20 | MFN2 | SERINC3 | PAN3 | MN1 |
| MBTPS1 | BACH1 | EPHA7 | ZNF654 | SORT1 | CXCL5 | SLC25A53 | SNX19 | EFS | DFFA |
| PRMT2 | FAM126B | SP1 | ATN1 | CAPZA2 | TUBA1A | AKIRIN2 | CRTC3 | ZBTB14 | N4BP1 |
| ELL2 | CABLES1 | LAMB1 | PRKAB2 | HYPK | ZCCHC14 | MAFK | CREB3L2 | CXCR1 | EIF2A |
| SMOC1 | ATP9B | GSTA4 | MT1A | SLC15A4 | ARNT | RFC1 | ARR3 | IST1 | DICER1 |
| ZNF45 | GRB10 | ZC3H7A | BIRC2 | UBR4 | HIC2 | MLLT10 | CRADD | PIGK | NDUFAF4 |
| NLE1 | DUOX1 | TRAF3IP2 | OSMR | CHP1 | FLT3LG | SREK1IP1 | GCSH | MAPK14 | CAMK2G |
| FDFT1 | RAPH1 | RAPGEF4 | CHM | ABR | ELF2 | ARNTL | TENT5A | GAB2 | AGFG1 |
| HECTD3 | EIF2D | CD55 | DYM | MAP2K3 | FKBP9 | RB1 | BTN2A1 | RCL1 | STXBP5 |
| HMGN3 | BTN3A3 | SLC38A2 | LXN | RAB3GAP1 | PCDHB13 | MICA | DSG3 | RANBP6 | LIMS1 |
| ENOSF1 | PDP1 | SPRY4 | DRAM1 | IGF2R | ZSCAN18 | EGLN1 | NMBR | TNRC6A | WTAP |
| NTAN1 | ETF1 | BCL2 | ZFC3H1 | SIRT3 | HIF1AN | PLAT | ERCC5 | TLR2 | RAP1B |
| ZNF75D | FBXO42 | HERPUD1 | NFYB | ACAP2 | DUSP8 | LSS | TMEM159 | COG6 | IFT57 |
| IL17RA | TBPL1 | FST | CDK14 | DOK4 | KYAT3 | STK17A | IDH3A | PALD1 | OGDH |
| TNFRSF10B | MBD2 | EIF5 | FBXW11 | WWC1 | MAP2K1 | PNMA8A | RAB9A | SLC39A14 | TNFRSF1A |
| MAPK8 | PRKAR2A | TSC22D2 | MYO5A | EP300 | YTHDC1 | BCLAF1 | THUMPD1 | MTMR2 | LIMK2 |
| KLHL9 | EHD4 | TAF1C | NCF4 | NEIL1 | NCK2 | RPS18 | PRKAR1B | WDR7 | CAP2 |
| RPL7 | EIF1AX | SPTAN1 | MPZL1 | ANXA2P2 | TMEM120B | MAPK1 | MED12 | SAMHD1 | PCM1 |
| AGO1 | AKAP10 | MTHFD2L | KRR1 | RRAGA | RAB21 | CAMKK2 | UIMC1 | FCGBP | IMPACT |
| CCNT2 | PRPF38A | SYNRG | FHL2 | TRIM8 | GLUD1 | SLC24A3 | TRIO | NCKIPSD | GPR3 |
| TESK1 | TCF25 | SSBP3 | PLK2 | PHF21A | LITAF | PPM1A | CAVIN3 | IGFBP4 | RPS27AP11 |
| GTF2I | UBR2 | PGPEP1 | SUCLG2 | PKIG | SOWAHC | ZNF274 | CRLF2 | SNAPC1 | ATPAF1 |
| PBXIP1 | SLPI | RWDD3 | ENPP4 | B3GLCT | GGCX | SSH1 | NFIC | CERK | ANAPC10 |
| ACTG1P10 | PRRX1 | LMNA | R3HCC1 | TIAM2 | XPO7 | MLLT6 | SBF1 | PCYT1A | TNFAIP3 |
| GATB | KAT5 | PML | PPP3R1 | NF1 | ANXA2 | ZFR | IP6K2 | MIS18BP1 | MAP4K4 |
| TEX10 | TUBA8 | CSAD | RTL8C | ACHE | PPP2R5B | ATF1 | APPL1 | CTDSPL2 | CIZ1 |
| HDHD2 | RPL36AL | SLC25A6 | PERP | RHOB | MYH9 | CYLD | TCEAL9 | VSIG10 | IRF4 |
| MED13L | SCARB2 | ANKRA2 | GGNBP2 | SF1 | SNX7 | MBTD1 | GOLGA4 | TP53I3 | PDLIM5 |
| ULK2 | AFAP1 | PNMA2 | TNFSF14 | HIVEP3 | EP400 | OXSR1 | ACTR1B | SERTAD2 | GLS |
| DNAJA2 | URB1 | HAUS2 | RASA1 | GNA11 | CD58 | STEAP3 | APIP | IRF2 | NRP2 |
| CIDEB | GPN3 | TMEM30A | ZNF84 | MGAT1 | CNOT7 | SYNGR1 | ZNF529 | WAC | S100A3 |
| MTX3 | PIR | ZNF827 | GALK2 | ATF4 | LNPK | FUT11 | CHKA | ODC1 | PCNX4 |
| ATF7IP | CNN2 | RASSF1 | RIPK1 | TWISTNB | ZNF226 | ASAP2 | NAP1L1 | PLCL1 | NQO2 |
| ADAM17 | SNHG7 | SERPINI2 | PPP2R2A | NUDT15 | ELP5 | ZNF337 | PDCD6IP | TMEM50B | RPP30 |
| UBC | CTNS | SYPL1 | CREBBP | TULP3 | DLAT | F2RL1 | BCR | HOMER1 | PIP4K2B |
| ZNF451 | HTRA1 | MAPK7 | GLRB | NADK | HHEX | NPC2 | SRRM2 | RNMT | FRRS1 |
| SP100 | FLII | CLK3 | MMP7 | RNASEL | PEX12 | PPP1R3C | AGPS | MTRF1 | ZP1 |
| NFKB1 | UBOX5 | PITPNB | DECR1 | ADAM10 | GABARAP | PPA1 | COL27A1 | MARVELD1 | B3GNT2 |
| ATG9A | MMP2 | CMAHP | COX4I1 | PNMA1 | CTNND1 | KCNJ14 | RPA1 | PTGER2 | ARPP19 |
| MARCKS | KDM4C | MAFB | TMEM128 | FCHSD2 | FAM107B | RGS12 | BIRC6 | TENT4A | REEP2 |
| ATR | ZFX | NDST2 | TNFRSF10A | SUSD1 | INSR | GTF3C3 | IER5 | MGST2 | DYNC1H1 |
| CDR1 | PRPSAP1 | PLXNA1 | BNIP3 | ZC3H12A | YPEL1 | ARFGAP3 | CCNY | THAP12 | IL6ST |
| CDC42EP1 | CARD16 | MAGEH1 | BTG3 | AMD1 | ITCH | RPH3A | ACTRT3 | MT-CO2 | PLXND1 |
| LSAMP | TBC1D9B | SPG7 | PHF13 | DDX18 | ZFP91 | CLIP2 | ZNF44 | C1orf21 | CEBPB |
| KRT23 | LAMB4 | NXN | PCMTD1 | PRELID2 | DNAJB9 | TADA2A | APBB2 | DSC2 | KTN1 |
| PITPNA | HDDC2 | TRIM13 | TOX | KCP | TSKU | NAA80 | TAF1D | CDV3 | MLLT3 |
| GLTP | RIOK3 | ZNF177 | EXOSC8 | ANKRD44 | RWDD2B | TRAF3 | MRPL46 | PRF1 | RUNX3 |
| PDE4D | ACSF2 | ALG13 | MAP3K7 | STEAP2-AS1 | ABHD14A | TAS2R14 | ZNF114 | STX16 | MAPK3 |
| SNORD123 | UBA7 | RNF169 | IFT46 | GPR161 |  |  |  |  |  |

|  |  |  |  | **Up-regulation** | **（N=1932）** |  |  |  |  |
| --- | --- | --- | --- | --- | --- | --- | --- | --- | --- |
| ATP2A2 | ACSL3 | YIPF5 | CTNNA1 | RELA | SMNDC1 | HMOX1 | MOB3A | VPS29 | CIAO1 |
| TRIP4 | MICU1 | PFDN1 | PI4K2A | GYG1 | GMCL1 | AKAP8 | UBA3 | LRRC47 | ACIN1 |
| ANKLE2 | MED15 | AMBRA1 | WASL | NSFL1C | ACTR6 | GOSR2 | CINP | BET1L | ZNF398 |
| NOL8 | CELF1 | PPID | TTC1 | STK25 | AGPAT1 | TAF9B | POLR1D | PPP4R1 | PSMD6 |
| SRPRA | AP3B1 | CCT4 | PAPOLA | ACTR1A | PLD3 | NCOR2 | ZKSCAN4 | ATP6V1B2 | GTF2E2 |
| OSTF1 | HNRNPD | MAGT1 | TOMM20 | CDK9 | ZBED1 | CDK11B | TNPO2 | FTH1 | DR1 |
| STX5 | SLC38A6 | RAC1 | RCOR1 | SLC30A9 | IGFBP7 | ZSCAN9 | RPARP-AS1 | KANSL2 | SNAPC5 |
| PRPSAP2 | IFNGR2 | ASCC2 | STAMBP | RNF10 | KPNA1 | RMC1 | RAB42 | XRCC6 | VHL |
| CASP8 | TMCC1 | GPR107 | SS18 | AREL1 | FAM214B | IL13RA1 | GMFB | MLH1 | EMC7 |
| DHX29 | PMEPA1 | MTMR14 | C2orf68 | OMA1 | MEF2D | PPRC1 | INIP | UBE2D1 | MTMR1 |
| HTATSF1 | PARP2 | TSEN2 | OSER1 | NEK4 | CDC42 | DNM1L | KDM2A | TRAPPC2 | ATP5MC3 |
| ARMCX6 | NDUFA5 | MRPL40 | KIN | MED10 | MLEC | SNX4 | STK10 | FASTKD5 | HSPA1L |
| LRRC41 | HBS1L | TXNL1 | CHST14 | STIMATE | ITGB5 | CDIPT | TARDBP | CPT2 | CAPNS1 |
| BCS1L | TRPC4AP | EBPL | COX7C | METTL2B | AK2 | AK1 | HTRA2 | AIMP1 | ACVR1B |
| CTSZ | EDC3 | TSPAN9 | HDLBP | PACS1 | EXOSC9 | TMX1 | MBTPS2 | TMBIM4 | AFG3L2 |
| PRMT5 | CCDC6 | BTBD10 | EXOSC2 | SPAST | UBA6 | RAD23B | NAA35 | MAP3K11 | GTF2F1 |
| PPA2 | USP46 | RAB35 | ZBTB6 | PSMF1 | UBE2E1 | ITGA5 | NIF3L1 | SGF29 | UPRT |
| SLC38A10 | BRD3OS | FKTN | ZNF496 | ARID1A | BCL2L11 | RABGGTA | TMED7 | SNX10 | RALA |
| ZMIZ1 | DYNLL2 | NEK6 | SNU13 | EWSR1 | TMTC3 | CDKN1B | UBE2H | ATP13A3 | TPM4 |
| ZNF184 | COA7 | CUL2 | XRCC5 | ACOT13 | SAP30BP | CHCHD4 | C20orf27 | RSAD1 | MVK |
| NPM1 | PHF5A | AMPD3 | KIAA0930 | BRD3 | TPD52L2 | TAF13 | ZRSR2 | PRDM1 | KIAA1958 |
| NECAP1 | SPATS2L | BFAR | TDRD7 | TRAM1 | RDH14 | IKBKG | TOMM22 | ARPC2 | UTP11 |
| BCAP31 | RPE | IFT27 | TAF1A | DHFR | CANX | PCYT2 | RABGEF1 | AMPD2 | TMEM30B |
| HSPA1B | TOP3A | EIF2AK2 | NUP188 | GZF1 | TMEM9B | SCYL2 | CLEC16A | PDCD11 | CEP170B |
| ARHGEF2 | GTF2H2 | ATP5MD | CAP1 | C16orf87 | DMPK | VEZT | CBWD1 | PSMD5 | COL5A3 |
| SH3BP4 | UBB | RRP15 | EPHB4 | APEX1 | TMBIM6 | MKRN1 | NCL | PPP2R5D | WBP1 |
| DUT | PAIP1 | DNASE1L1 | NPEPPS | HMGB1P5 | UBL7 | GPN1 | CUL4A | GSK3B | SFPQ |
| NBEAL2 | PSMC6 | EIF4E | ANKRD9 | LCORL | QRSL1 | PPP1R7 | RAB20 | WDR12 | ATF6 |
| TRADD | PON2 | PSMB1 | UMPS | NCF2 | CUX1 | RNF4 | NKRF | LAP3 | UTP14A |
| PDLIM2 | SRP19 | GALNT1 | EHD1 | LARS2 | BID | DIABLO | MSRB2 | IMP3 | AGPAT5 |
| ZNF324B | PXDN | PGM2 | PI4KB | SMIM10L1 | RRP1B | CCDC47 | SLC10A3 | ARID3B | DNAJB2 |
| RBM14 | SNAP29 | CCDC85C | GEMIN2 | STK4 | ABCC10 | PSMD9 | ELOVL1 | MDM4 | ANAPC15 |
| LBHD1 | WDR45B | PSMA1 | GDI2 | SEH1L | PAK2 | CCP110 | DBNL | SLC31A1 | PPARD |
| NUDT3 | MRPS33 | NUP43 | KPNB1 | FANCL | NIT1 | SLC35E3 | SPARC | GLRX3 | TIGAR |
| PRRC2C | OTULIN | SRSF2 | NUP58 | C1orf216 | FAM114A1 | PSMB6 | MYO9B | NOL3 | CARS2 |
| SLC6A6 | SEC23IP | PLAGL2 | TMEM259 | CASK | NDUFA12 | SPG21 | FAM210A | CDK8 | DHX9 |
| IRX3 | GNA13 | PANK3 | ARL1 | PNPLA6 | DLGAP4 | SLC37A4 | SLC25A32 | PATJ | SNRNP40 |
| SLC35E1 | POLR3C | PCMT1 | CSPP1 | INTS1 | TWF1 | HMGCS1 | TMA16 | PSMB7 | PA2G4 |
| TICAM1 | CENPQ | SAT1 | TYK2 | DCAF6 | ATP6V1A | TRIM27 | MINPP1 | GRSF1 | NSL1 |
| ZNRF1 | PFDN4 | INTS14 | MAP3K13 | RUBCN | ZNF101 | TATDN2 | NDUFB1 | SUB1 | CBFB |
| PLXNB2 | PRPS1 | UQCRC1 | PSME3 | BCAR1 | POLR3E | KDM5C | GSTO1 | RIT1 | SEPHS1 |
| HEXIM1 | EMG1 | KDM2B | USP1 | CCZ1B | TMEM184B | GOLGA2 | SLC7A1 | LILRB2 | PSMD13 |
| CLCN5 | GALNT18 | ACYP1 | FBXO28 | LIN37 | SQSTM1 | RTCA | ABCF2 | HDHD5 | UBE2D2 |
| CLTB | WDR74 | BSCL2 | TMEM65 | NAGPA | DNM2 | PGAM1 | RABGGTB | POLM | ME2 |
| POLR2D | SNRPA | HEATR1 | MRPS17 | PPIC | VMAC | RAB34 | ATP6AP2 | ADCY9 | SNHG12 |
| ATP1B3 | ABCB10 | PGM3 | CHERP | FAF2 | RRM2B | SMYD2 | WIPI1 | DDIT4 | USP7 |
| ULK1 | MED26 | PTGFRN | TXNRD2 | DDX23 | MDM2 | S100A13 | RPS6KC1 | BTK | BNIP1 |
| IPO13 | EVA1A | ADAMTS13 | CISD1 | ELMO2 | COL6A2 | PABPN1 | EIF2B5 | NQO1 | PLA2G7 |
| HSP90B1 | CDK5RAP3 | GTPBP2 | WFS1 | ILVBL | PCBD1 | SPSB3 | ARHGAP8 | MAPK9 | MRPL34 |
| RIC8A | SUMO3 | MAPKAPK3 | DCTN5 | CAD | POLR2L | AKIP1 | PSMD1 | NASP | KIAA2013 |
| SPTSSA | ANP32A | DDX54 | RCN2 | EEF1D | DYRK2 | GRWD1 | RER1 | YWHAB | MSMO1 |
| TGIF2 | PLEKHA8 | RNF19B | SLC39A13 | ILF3 | GLMN | NIT2 | PIM3 | DPAGT1 | FAM53B |
| LSM3 | PTTG1IP | HSBP1 | NMI | NDUFB6 | WDR77 | IDE | NRAS | BHLHE40 | BLZF1 |
| TDG | NARS2 | TCEA1 | MRTO4 | TMEM102 | CPNE3 | CD2AP | SOAT1 | UFD1 | GLRX |
| IKBKB | DEDD | CCSAP | ING2 | VCP | LUC7L | SLC22A5 | MLF1 | SRM | NINJ2 |
| LIMD1 | SERP1 | NUBP1 | GART | DPM1 | BZW1 | DUSP5 | AP2B1 | ZNF174 | SLC25A29 |
| ZNF629 | PSMC2 | HSP90AA1 | PMPCA | PPP1R10 | NUPR1 | ATP5MPL | PIAS3 | SPATA2 | STK11 |
| NARF | IGSF6 | GDF11 | ELF3 | NCBP2 | LYPLAL1 | RPL8 | FCER1G | PTP4A2 | TXNDC9 |
| RNF114 | LRIF1 | APOL2 | PSME4 | FBRSL1 | FLNB | TSPO | SPATA2L | CHD4 | PEX13 |
| CENPB | FBXL8 | CCDC86 | SLC25A14 | BCOR | PRDX5 | ARSB | SRPK2 | MSANTD3 | MRPL57 |
| CDH1 | CASP2 | GNPDA1 | CEP83 | RNF149 | LACTB | LSM5 | DNPEP | DNAJC5 | C1QBP |
| VBP1 | CHRAC1 | PSMA2 | NFS1 | SOD1 | SETDB1 | TMEM33 | HSPA9 | NEDD8 | CAPN1 |
| UBALD2 | NUP205 | PIH1D1 | ZNF512B | UBE2I | PARS2 | LCP1 | PPP1R11 | KIAA0513 | PLEC |
| TOPBP1 | SIPA1 | GPR35 | RBCK1 | NAGA | MIPEP | COPS6 | ORAI3 | UROS | MORC2 |
| PSMA3 | HDAC1 | EXOSC1 | RNF34 | BOLL | MVD | TGIF1 | SLC9A3R2 | RNF126 | IFITM3 |
| DNAJC2 | PSMC3 | TIPIN | PNO1 | GBP3 | APTX | LPXN | VAV1 | SPHK2 | WFDC1 |
| NADSYN1 | SCAMP4 | BCAT1 | GINS3 | RAD1 | STAU1 | PSMD8 | HSPA13 | DOLK | GLYR1 |
| PLEKHB2 | COQ2 | PLP2 | RRP9 | SCLY | UAP1 | VANGL2 | CYTH2 | PSMD10 | ZNF544 |
| BAZ1A | NBL1 | NET1 | VPS16 | SKIV2L | CTSB | VRK1 | NEMP1 | NOLC1 | USP39 |
| LAT | COL18A1 | OCEL1 | CRTC2 | MARK2 | SRA1 | MSH6 | RBKS | APBA3 | CD53 |
| TNFAIP6 | RAB1B | EIF2S2 | NUDT18 | SLC26A1 | EFR3A | BRCC3 | GTF2E1 | PSMB2 | CATSPERG |
| BCL2L1 | RABEPK | PTK2 | NSDHL | LGALS8 | ZMPSTE24 | HNRNPU | HCK | TLNRD1 | DAP |
| MDH2 | HMGN2 | ARHGAP4 | LARP4 | MRPL37 | IRF3 | ING1 | COA3 | MCRIP1 | BLVRB |
| ELK1 | NPRL2 | POR | PFKL | SRGAP2 | ACP1 | CDK2AP1 | NIPA1 | SLC6A8 | DSP |
| DNAL4 | CLPB | GNAS | DYRK1B | PDCD10 | MAPK8IP3 | GPRC5C | PRKCD | DNAJA1 | TP53BP2 |
| CCDC9 | ARPC5 | NAPA | LDHA | DCAF7 | CNOT3 | SMC2 | NOP58 | IDUA | SRSF1 |
| SIGMAR1 | SPATA20 | MCTS1 | GALNT2 | BAG1 | MYO10 | RGS5 | NBDY | ATXN2L | GPI |
| C8orf33 | SH3BGRL3 | MCUB | ICAM1 | PSMC5 | HEATR3 | TP53TG1 | RPUSD3 | EVI2A | PRMT1 |
| HNRNPA2B1 | GRIP1 | TUBBP1 | HSPA1A | PLXNC1 | GALNT10 | DGCR6L | DYNLL1 | BAD | TMEM186 |
| HK1 | LDOC1 | LRRC42 | NCAPD3 | CLDN4 | NOSIP | FLOT1 | NOL11 | CHRNA1 | MYD88 |
| RFC5 | FYB1 | YWHAH | RGS16 | TEX30 | PDCD4-AS1 | EMILIN1 | EEF1E1 | PSMG1 | MX2 |
| LSM2 | APOL1 | APRT | HSD3B7 | UBE2V2 | FTSJ1 | ZMIZ2 | ADCK2 | MGAT2 | RBX1 |
| GRK6 | CARM1 | TGM2 | SNRPD3 | RRM1 | AKT1S1 | SSBP1 | HAX1 | GSS | SLA |
| AP1G2 | AP3M2 | SLC25A15 | HSPA4 | PHTF2 | PSMB10 | SLC3A2 | SLC38A1 | STK39 | KCTD3 |
| POP4 | ZNF581 | NDC1 | NCS1 | CDC34 | ARPC4 | UCHL5 | PTBP3 | GEMIN6 | NT5DC2 |
| TOR1AIP2 | ENDOG | MYCBP | CYP51A1 | AGRN | SRRT | TMEM51 | CNOT11 | HPSE | SLIRP |
| MACO1 | GSK3A | RPP40 | NOCT | FAM207A | THAP8 | SMPD4 | ANKRD39 | LMO7 | PAXIP1 |
| CAPN12 | CD2BP2 | HSPD1 | PLOD1 | SMOX | TBCD | B4GALT2 | LOXL2 | AUP1 | NUP85 |
| DBN1 | ACTG1 | RHOC | AKT1 | BCKDK | LUM | CDC42SE1 | COL6A1 | FAM136A | EAF2 |
| HLA-DRB1 | ARF3 | DAXX | RRBP1 | SQOR | RIOX1 | FBRS | CAPZA1 | CYB5R1 | VDAC1 |
| DDX11 | PHB | TMEM41B | SLC35F6 | NAT10 | SRP54 | TFDP1 | SEC11C | NFKB2 | DHX58 |
| TSFM | GLB1 | LONP1 | TUBA1B | TFG | OVGP1 | DPP7 | MAST2 | GLA | TMEM254 |
| UBE2F | PSMD2 | UBFD1 | PDCD6 | UBE2O | STX3 | SLC25A44 | VKORC1 | ACAT2 | ZMYND8 |
| KRAS | SPHK1 | ST6GALNAC2 | HMBS | KIAA0895 | DYNLRB1 | GTPBP4 | CPNE1 | MVP | DKC1 |
| LETM1 | CHST15 | PSMA4 | ARFIP2 | FUS | GOT2 | MRPL52 | URB2 | KCTD15 | TPI1P1 |
| BIRC7 | PRPF19 | BLVRA | DDX41 | TIMM23 | SH3GL1 | EML2 | SRSF9 | HILPDA | TRPC4 |
| NTMT1 | MPHOSPH9 | GPC1 | EIF5A | AIFM1 | NINJ1 | SPOCK1 | QSOX1 | SCNN1D | PPT1 |
| FJX1 | MCM5 | TMTC4 | SBNO2 | BDH1 | ITPR3 | ITGB2 | PFN2 | CYCS | NDUFA2 |
| PSMA6 | RAN | POLE | HNRNPA3P1 | CASP3 | FAM91A1 | PPME1 | ERO1A | SH3BGRL | MTHFD1L |
| PRKCZ | ARPC3 | PDK3 | ARHGDIA | AZIN2 | HACD3 | SOCS1 | PSMD12 | DAPK3 | YKT6 |
| IRF1 | HYOU1 | NOMO1 | KDELR2 | TRIM14 | FDPS | ZNF217 | NOP56 | ICA1 | PLEKHO1 |
| IL1RN | ARHGAP45 | MOGS | DENND2D | PSMD11 | COTL1 | POLD2 | PSME1 | HCFC1R1 | ITGB7 |
| PARN | ENOPH1 | ENPP1 | HSF1 | PPP1R9B | ZNF688 | PIPOX | MCHR1 | P4HA2 | B3GALNT1 |
| PTOV1 | LASP1 | ARMC6 | FAM89B | RNPS1 | RPN1 | SNX24 | ACBD3 | SEC13 | GPS1 |
| ZNF764 | GLO1 | RAI14 | CD48 | RFC3 | FAM162A | PLOD3 | MEN1 | PSMB5 | ALDH1B1 |
| LGALS3BP | ZNF623 | CELSR2 | GRB2 | CASP6 | SEC16A | WDR4 | LMX1B | C16orf74 | DNMT3A |
| MGAT4B | CHCHD2 | PHTF1 | PIDD1 | SKIL | XPO5 | TRMT12 | MAPK13 | SYTL2 | SLC39A1 |
| EBNA1BP2 | DDX49 | PPM1G | MASTL | HMOX2 | PDZD11 | NCLN | ZNF771 | LDLRAD3 | DDR1 |
| IGFBP2 | HSPA5 | RBM11 | OPN3 | NETO2 | HLA-B | MTSS1 | MSRB1 | HRAS | AIMP2 |
| AL109918.1 | RPA3 | TMEM106C | IRF5 | CTTN | AMDHD2 | CNIH4 | NOP16 | C11orf80 | CEP250 |
| VEGFA | COLGALT1 | RAVER1 | BUD23 | PIK3R3 | FANCG | METTL1 | RANBP1 | CLNS1A | ERGIC1 |
| NDUFS2 | OGFR | PSMA7 | RGPD4 | IGSF3 | GCH1 | GEMIN7 | CCT6A | GAMT | CCNB3 |
| SIDT1 | UBAP2L | CD86 | SNRPA1 | PDCD2L | SEM1 | SLC19A2 | SOX4 | RNF213 | FGD6 |
| RNASET2 | PPAT | ENSA | SLC26A6 | SFN | PPP6R1 | TUBB | SNRPD1 | TRAF5 | RPN2 |
| NFKBIB | PSMB8 | SAMSN1 | WDR18 | PSEN2 | FAM174B | HSPH1 | HLA-F | MAGOHB | NRARP |
| NFATC4 | EIF4A3 | CSTF2 | MFSD10 | STX6 | SPDL1 | PUS1 | CYP2S1 | PUF60 | NHP2 |
| RITA1 | CSE1L | PDSS1 | PYCR2 | RAC2 | IPO4 | IRAK1 | PDIA3 | PARD6A | F2R |
| PSMD14 | COX6A1 | COPA | PSMD4 | DENND1C | IMPDH1 | SERF2 | LIG3 | COX17 | HLA-A |
| ATIC | TGFB1 | YWHAZ | PPIF | NCAPD2 | MEX3D | SH2B2 | RELB | ANP32E | MTERF3 |
| PGK1 | DOHH | PHEX | PWP2 | NT5M | DNAJC9 | ENAH | PTK7 | TCF3 | MRPL15 |
| OCLN | PIM2 | RRP1 | UGDH | CORO1B | MFSD2A | TPBG | SERPINE2 | CRIP2 | PSMA5 |
| FHAD1 | SPTBN2 | TRAPPC6A | IGF1R | MCM3 | BZW2 | IFIH1 | ABHD3 | DECR2 | MTX1 |
| TRAF3IP3 | STK26 | EGLN2 | AXIN1 | GALK1 | DAP3 | LGALS9 | ZNF124 | FANCF | CD9 |
| FAM110B | GTPBP3 | C2orf15 | JMJD4 | ATP5MC1 | BMF | MAPT | SELENOH | PSMC3IP | POLA2 |
| ABCG1 | FBXO5 | TUBB4B | CDCP1 | IFI44L | ST3GAL1 | PARP12 | TCEA3 | CYCSP55 | PPIAL4A |
| CHEK2 | SHMT2 | P3H3 | NDUFA13 | TGFB3 | CIART | GLRX2 | RFX5 | ADAR | DHCR24 |
| PTP4A3 | NOP2 | CD22 | PSMB4 | TMEM147 | NDE1 | CD276 | ZNF93 | ABCB9 | LCK |
| CYBC1 | TMSB15B | TRAF4 | SERPINH1 | TXN | PKM | RANGAP1 | DPM3 | CCDC28B | NCDN |
| PPP1R16A | VPS72 | GTF3C1 | SRD5A3 | GLIS2 | SNRPG | PAICS | B3GNT4 | MAD2L2 | RUVBL2 |
| COL13A1 | RGS14 | SMPDL3B | SLC35B1 | LSM7 | CACYBP | POLD1 | MYB | GSR | P4HB |
| ISYNA1 | UBQLN4 | BRMS1 | HMG20B | CCL5 | MFSD12 | RANBP17 | NOX1 | TNFRSF12A | HSPBP1 |
| ADRM1 | LURAP1L | E4F1 | TPI1 | TMSB10 | KDM5B | IRF9 | SHB | CRELD2 | SLC1A4 |
| BYSL | ASNS | PTRH2 | ITGA11 | SCO2 | TBC1D9 | AMMECR1 | WDR54 | CKAP4 | TRABD |
| ELOC | NRSN2 | VWA1 | ALDH18A1 | MAP4K1 | FHDC1 | DPCD | PSMC4 | P4HA1 | LILRB4 |
| GPATCH2 | PSMB9 | DPM2 | NME4 | REC8 | LIG1 | SERPINE1 | NDUFS6 | STIP1 | RUVBL1 |
| PRELID3A | ARL6IP1 | VAMP8 | IFIT3 | RPP25 | RCC2 | PNP | BCL3 | HLA-DQB1 | PDXP |
| TANC2 | PRSS23 | BAK1 | IFI35 | TOR3A | POLR2H | RFC2 | TELO2 | LSM1 | ZNF48 |
| SLCO5A1 | MCM6 | ALDOAP2 | RHBDD3 | GSDMD | MRGBP | TRERF1 | SLC19A1 | BUB3 | SEMA5B |
| RAD21 | APOC1 | EFNA1 | LRRC61 | CENPS | IFI30 | KLC2 | IFI44 | DNPH1 | MAGED1 |
| PPEF1 | GAPDH | COPE | SYT7 | IFIT2 | THOC6 | POP7 | ST3GAL4 | CERS6 | SEC61G |
| CORO1A | UBE2L6 | CAPG | KNTC1 | ZNF668 | CXCR4 | HPRT1 | NUDT1 | DTYMK | SAP30 |
| ALDOA | TBC1D30 | ARPC1B | CHAF1A | CCDC137 | IKZF3 | MAPKAPK2 | ZWILCH | C1QL1 | FADD |
| SMS | MIR155HG | GK | ATP7B | PSPH | ISG20 | MYH13 | TP53INP1 | EBP | BRCA1 |
| IFITM10 | GTF2IRD1 | EPHB2 | TFRC | HLA-G | CSPG5 | TIMP1 | PDRG1 | RRS1 | ASPSCR1 |
| NFKBIE | PLEKHF2 | PPP1CA | TPM3 | SLC20A1 | BAX | ELOB | PHKG2 | DHRS13 | BARD1 |
| DHCR7 | PDIA4 | TEAD4 | THBS2 | TUBG1 | KDM4B | HMGB2 | SIGIRR | PSMG3 | SMIM29 |
| TIMM17A | PSMB3 | TRAF2 | CDC25B | STAP1 | PPDPF | WDR90 | KDELR3 | AGRP | PFDN6 |
| ABCA12 | SDSL | IFITM1 | JAKMIP2 | SLC25A10 | S100A11 | TRPS1 | ZNF239 | DBF4 | AEBP1 |
| SREBF1 | NCAPG2 | PAK4 | DENND1B | TIMM17B | SEPHS2 | CCT3 | DOP1B | STARD3 | ATP6AP1 |
| SKAP1 | AP1S1 | SSX2IP | MTFP1 | TOMM40 | PARP1 | PFDN2 | MAGED2 | CDCA4 | DHRS2 |
| IL18 | PSME2 | TCF19 | RHOF | SNRPB | REEP1 | DLEU2 | UGCG | MRPL13 | BASP1 |
| BCL2L12 | STAT1 | NFE2L3 | MRPS12 | FDXR | WDHD1 | TBX1 | CYB561 | TMEM45A | IER3 |
| NR2F6 | LPCAT1 | NREP | PFKFB4 | PAGR1 | FLAD1 | TTYH3 | ATP4B | LRRC59 | GOLM1 |
| PPFIA4 | SP140 | MAZ | LAPTM4B | CDK5R1 | JPT2 | COLEC10 | MMP8 | IDH2 | EPCAM |
| MMP14 | MZT2B | SPINT2 | GDNF | CPN2 | ABRACL | MRPL14 | C1orf112 | TNFRSF4 | CTPS1 |
| CTSD | NVL | SPAG1 | ANGPTL6 | SLC27A2 | TTC39A | BBC3 | EFR3B | CCND1 | VAV3 |
| PLAU | C4B | CENPN | MICB | FZD2 | MDK | RPL39L | LMNB2 | STXBP2 | SYCP2 |
| BST2 | PVT1 | MARCKSL1 | PCNA | CDK5 | MBOAT2 | LSM4 | ACOT7 | GADD45G | MBOAT7 |
| LIME1 | MRPS34 | TUBA1C | EIF4EBP1 | QPRT | ETNK2 | FKBP4 | SPDEF | PSMD3 | COL3A1 |
| EME2 | PHLDA2 | MCEMP1 | SDF2L1 | MTHFD2 | RIMKLA | MRPL12 | CD24 | EXOSC4 | LOXL1 |
| SAC3D1 | SLC2A1 | LINC01711 | MTCL1 | FER1L4 | DDX39A | OAS1 | SMC4 | ENO2 | RND1 |
| RAD54B | POLE2 | C3orf52 | HMGA1 | DOC2A | CHEK1 | LSR | RASGRP1 | CDH11 | INAVA |
| TIMELESS | ZNF296 | BIK | CXCL13 | SPAG4 | BOP1 | ESRP1 | RP1 | POLR3K | TAP1 |
| PPP1R14B | MIF | EGLN3 | PARPBP | IL11 | COL12A1 | TMEM158 | KRT18 | ATP2A3 | TSPAN13 |
| FADS2 | FAM234B | PRRT2 | C5orf49 | ROCK1P1 | HSPB1 | PAX9 | ADORA2A | TRIB3 | FEN1 |
| COL1A2 | LIMD2 | SH2D2A | LRP8 | RASSF7 | SLC2A6 | BCL2A1 | VCAN | PCSK9 | HSPA6 |
| F2RL2 | SLC35D3 | RBPJL | PTPRN | ESR1 | SIAH2 | HELLS | CA12 | OAS2 | ZNF367 |
| NEFH | SIX4 | ADAM19 | FAM83H | ATG9B | PLAUR | DNA2 | PANX2 | IER5L | CCNF |
| PLK4 | CDC7 | MYCN | TCL1A | MX1 | MCM2 | MCM4 | GATA3 | ESPN | PMAIP1 |
| STMN1 | C2orf50 | PYCR3 | TSTA3 | RLN2 | SLC52A2 | RGS4 | TMEFF1 | SOX12 | C15orf48 |
| N4BP3 | SLC9A3R1 | PSRC1 | ERBB2 | EFNA3 | TPD52 | LTA | GALR2 | COL5A2 | KRT19 |
| SDC1 | KLHL35 | GPR84 | PRR7 | HES2 | B4GALNT4 | NUP210 | LAMP3 | FANCA | NEFL |
| SLC39A4 | KCNK1 | ECT2 | PYCARD | ECE2 | NME1 | RACGAP1 | CEACAM7 | IFI27 | IRF7 |
| ATAD2 | FANCI | HS3ST3A1 | ASPN | FBXL16 | UNC5B | COL5A1 | LAGE3 | LEF1 | HOXB1 |
| TYMS | CHST1 | MIEN1 | ADGRB2 | TH | STIL | IL9R | CRABP2 | GINS2 | FOXP3 |
| ERFE | MYBL1 | CDC25A | RNFT2 | MAD2L1 | KPNA2 | UTS2 | SLC7A11 | JPT1 | PADI1 |
| AUNIP | ORC1 | ARHGAP11A | RAD51AP1 | SQLE | MMP3 | UNC5A | SPP1 | CYP21A2 | CENPU |
| SLC16A3 | SRD5A2 | TACC3 | GPRC5A | GDF15 | FCAMR | OASL | HMGB3 | BGN | FGFR3 |
| LMNB1 | CDT1 | CCNE2 | PAFAH1B3 | SLC16A6 | MMP12 | CACNA1H | JPH3 | LY6H | GINS1 |
| PAX2 | KIAA0319 | OIP5 | EDN2 | MUC1 | KIF11 | TFF3 | CTHRC1 | ORC6 | UBE2S |
| CDH2 | CCNB1 | SLC7A5 | TRIP13 | ZWINT | RAD51 | SHCBP1 | PBOV1 | PAQR4 | KIF23 |
| PRC1 | E2F1 | HMGA2 | INHA | HOXB9 | ESPL1 | AMH | TERT | PRR11 | RAD54L |
| SPAG5 | CDKN2A | SPC25 | ERCC6L | TTK | CCNE1 | CCNA2 | ELFN2 | E2F7 | IFI6 |
| TRIM15 | CCDC185 | RAMP1 | HES6 | CDKN3 | SLC18A1 | CDC6 | FN1 | FOXD1 | RET |
| CCNB2 | HMMR | PIMREG | MT1H | DTL | TK1 | FBN2 | NUSAP1 | ASF1B | NCAPG |
| MKI67 | CDCA5 | KIFC1 | AURKA | CEMIP | MMP9 | CDK1 | MCM10 | SYNGR3 | ARTN |
| PTTG1 | NDC80 | UBE2T | SGO1 | CENPF | CLEC5A | BUB1 | DLGAP5 | CENPA | PCLAF |
| NELL2 | CADPS | KIF2C | UHRF1 | AURKB | SKA1 | SALL3 | KIF20A | GAD1 | ANLN |
| RRM2 | EXO1 | SOX2 | PLK1 | CEP55 | PBK | INHBA | FOXM1 | TOP2A | FAM83D |
| CDC20 | ACOD1 | HJURP | DIO1 | AOC1 | ASPM | ISG15 | BIRC5 | KIF18B | TPX2 |
| CXCL10 | MELK | NMU | TUBB3 | DMRT1 | CXCL11 | LHX9 | MYBL2 | KIF4A | INA |
| EPO | PKMYT1 | PITX1 | VSTM2A | IFNB1 | UBE2C | CEACAM6 | FABP6 | FOXG1 | CA9 |
| PLAC1 | DMP1 | PRAME | SYT13 | ASCL1 | CST2 | INSM1 | GRM4 | GABRA5 | MMP13 |
| PCSK1 | MMP1 |  |  |  |  |  |  |  |  |
