# Supplementary material for: Identification of a combined apoptosis and hypoxia gene signature for predicting prognosis and immune infiltration in breast cancer
Source: Cancer Med. 2022 Apr 20;11(20):3886–901. doi: 10.1002/cam4.4755 (PMC9582692; doi:10.1002/cam4.4755)
Supplement: Supplementary file 5 — Table S2 [file CAM4-11-3886-s002.doc]

Supplementary table 2. AHGs derived from univariate Cox regression analysis

| **Gene** | **HR** | **P-value** |  |
| --- | --- | --- | --- |
| RAD54B | 1.347187 | 0.011516 |  |
| SLC19A1 | 1.119669 | 0.011712 |  |
| GRIP1 | 1.094252 | 0.014181 |  |
| RAD1 | 1.086916 | 0.028248 |  |
| RAD51 | 1.065417 | 0.037154 |  |
| MCTS1 | 1.06199 | 5.60E-05 |  |
| NUP43 | 1.052783 | 0.00111 |  |
| CDK8 | 1.052341 | 0.027109 |  |
| ZNF623 | 1.050016 | 0.00204 |  |
| TMEM65 | 1.047931 | 0.028369 |  |
| AIMP2 | 1.046784 | 0.006128 |  |
| TXNDC9 | 1.045663 | 0.025861 |  |
| RNF4 | 1.044558 | 0.045679 |  |
| SLC25A32 | 1.044019 | 0.002425 |  |
| SCYL2 | 1.04015 | 0.028259 |  |
| CISD1 | 1.040083 | 0.003285 |  |
| PGAM1 | 1.03987 | 0.010437 |  |
| ELOC | 1.035007 | 0.001846 |  |
| PTK2 | 1.03399 | 0.007539 |  |
| AIFM1 | 1.032158 | 0.002111 |  |
| P4HA2 | 1.031837 | 0.003854 |  |
| FAM114A1 | 1.028608 | 0.006001 |  |
| QPRT | 1.028032 | 0.002068 |  |
| TAF9B | 1.025806 | 0.004525 |  |
| AGPAT1 | 1.024897 | 0.033441 |  |
| PCMT1 | 1.02478 | 6.81E-06 |  |
| PSMD10 | 1.019339 | 0.027358 |  |
| SHMT2 | 1.019189 | 0.005006 |  |
| TRMT12 | 1.018991 | 0.045529 |  |
| SH3BP4 | 1.018369 | 0.005857 |  |
| VBP1 | 1.018254 | 0.015681 |  |
| MRPL13 | 1.017776 | 0.000201 |  |
| VHL | 1.017538 | 0.011242 |  |
| CACNA1H | 1.016919 | 0.015403 |  |
| PAICS | 1.016663 | 0.000344 |  |
| C8orf33 | 1.016555 | 0.000744 |  |
| TUBA1C | 1.014772 | 0.010538 |  |
| MAGT1 | 1.01433 | 0.008419 |  |
| VDAC1 | 1.011244 | 5.21E-05 |  |
| HSPA4 | 1.010303 | 0.019667 |  |
| STIP1 | 1.009994 | 0.00186 |  |
| HSPA9 | 1.009105 | 0.000119 |  |
| IL13RA1 | 1.008818 | 0.010165 |  |
| ESRP1 | 1.007806 | 0.040784 |  |
| CPNE3 | 1.007763 | 0.014576 |  |
| PGK1 | 1.007672 | 3.57E-08 |  |
| TPD52 | 1.007508 | 0.003336 |  |
| PRAME | 1.006438 | 0.022152 |  |
| SLC38A1 | 1.00636 | 0.007337 |  |
| ATP6AP1 | 1.005501 | 0.02694 |  |
| YWHAB | 1.005432 | 0.000551 |  |
| KDELR2 | 1.005369 | 0.007944 |  |
| CCT4 | 1.005292 | 0.041663 |  |
| AIMP1 | 1.004265 | 0.002662 |  |
| BCAP31 | 1.003566 | 0.021587 |  |
| EIF4EBP1 | 1.002532 | 0.004043 |  |
| SDC1 | 1.002387 | 0.002446 |  |
| HSP90AA1 | 1.001149 | 8.88E-05 |  |
| CD24 | 1.000205 | 0.049401 |  |
| JUN | 0.995846 | 0.024744 |  |
| C1S | 0.9958 | 0.034072 |  |
| JUND | 0.99572 | 0.010933 |  |
| GSN | 0.989465 | 0.010054 |  |
| SOCS3 | 0.988796 | 0.014086 |  |
| CEBPD | 0.987803 | 0.029648 |  |
| SRSF5 | 0.986423 | 0.03637 |  |
| JAK1 | 0.98558 | 0.018612 |  |
| BTG1 | 0.98449 | 0.001787 |  |
| FZD7 | 0.9843 | 0.036519 |  |
| NFKBIA | 0.982896 | 4.52E-05 |  |
| ETS1 | 0.982871 | 0.04659 |  |
| SGCE | 0.982565 | 0.03537 |  |
| ERRFI1 | 0.981947 | 0.005695 |  |
| GBP2 | 0.980968 | 0.007121 |  |
| ARID5A | 0.978621 | 0.043477 |  |
| CSRNP1 | 0.977755 | 0.030056 |  |
| TNFAIP3 | 0.972695 | 0.043416 |  |
| GMFG | 0.972408 | 0.029105 |  |
| NFKBIZ | 0.972262 | 0.027028 |  |
| BTBD6 | 0.965985 | 0.003149 |  |
| AK3 | 0.965687 | 0.042211 |  |
| SERTAD1 | 0.958795 | 0.023134 |  |
| TBC1D4 | 0.957538 | 0.022961 |  |
| TNIP1 | 0.956147 | 0.000276 |  |
| UBA7 | 0.954701 | 0.003113 |  |
| PDLIM4 | 0.952678 | 0.011839 |  |
| BTN3A3 | 0.949283 | 0.045331 |  |
| DENND5A | 0.948306 | 0.047446 |  |
| POLR3GL | 0.946246 | 0.002295 |  |
| NEDD9 | 0.944811 | 0.01524 |  |
| SORBS1 | 0.942029 | 0.013277 |  |
| PRF1 | 0.937237 | 0.030736 |  |
| ZNF655 | 0.935653 | 0.032043 |  |
| MAFF | 0.933007 | 0.035382 |  |
| IRF2 | 0.922473 | 0.000155 |  |
| DDB2 | 0.913151 | 0.01858 |  |
| SAV1 | 0.910653 | 0.000894 |  |
| STOX1 | 0.909417 | 0.035203 |  |
| JAK2 | 0.907364 | 0.011924 |  |
| MYO15B | 0.906652 | 0.03469 |  |
| RCL1 | 0.892633 | 0.041323 |  |
| ENOSF1 | 0.887652 | 0.027639 |  |
| STX11 | 0.830746 | 0.005406 |  |
| BIN3 | 0.819653 | 0.013521 |  |
| BEND5 | 0.809468 | 0.00784 |  |
| STAT4 | 0.801781 | 0.029757 |  |
| MAP2K6 | 0.767587 | 0.017677 |  |
| ANKDD1A | 0.573534 | 0.021053 |  |
| FLT3LG | 0.481776 | 0.001353 |  |
| TSLP | 0.153394 | 0.005453 |  |
| CLCNKB | 0.095954 | 0.041944 |  |
| GUSBP11 | 0.086135 | 0.021417 |  |
| SLC47A2 | 0.01288 | 0.04782 |  |

Abbreviations: AHGs, apoptosis and hypoxia-related genes; HR, hazard ratio.
