# Supplementary material for: Identification of a combined apoptosis and hypoxia gene signature for predicting prognosis and immune infiltration in breast cancer
Source: Cancer Med. 2022 Apr 20;11(20):3886–901. doi: 10.1002/cam4.4755 (PMC9582692; doi:10.1002/cam4.4755)
Supplement: Supplementary file 6 — Table S3 [file CAM4-11-3886-s006.docx]

**Supplementary table 3.** Differentially expressed AHGs between high- and low-risk groups

| **Gene** | **FDR** | **Log2 FC** | **P-value** |
| --- | --- | --- | --- |
| CSN1S1 | 1.21E-10 | -7.265021639 | 1.53E-11 |
| KRT1 | 3.25E-06 | -6.458542042 | 9.40E-07 |
| LOR | 2.39E-13 | -5.816854414 | 1.75E-14 |
| SMR3B | 7.79E-13 | -3.886929616 | 6.34E-14 |
| SNORD15B | 4.41E-06 | -3.748441475 | 1.31E-06 |
| RNU4-2 | 0.002076498 | -3.546564264 | 0.001046294 |
| RNU1-67P | 0.020645331 | -3.475202913 | 0.013042103 |
| SCARNA6 | 0.00027937 | -3.426162082 | 0.00011793 |
| SNORA54 | 0.011763046 | -3.031986756 | 0.007015355 |
| SNORA74B | 0.008549618 | -3.001299126 | 0.004939963 |
| RNU5E-1 | 0.020905845 | -2.824928607 | 0.013222874 |
| ARHGAP36 | 3.50E-07 | -2.715538958 | 8.40E-08 |
| SCARNA21 | 0.003403568 | -2.535355504 | 0.001789867 |
| KRTDAP | 5.21E-05 | -2.443891922 | 1.91E-05 |
| SCARNA13 | 5.13E-06 | -2.318977582 | 1.54E-06 |
| SNORA7B | 0.002317849 | -2.126910538 | 0.001180296 |
| SNORA12 | 0.009306296 | -2.088399695 | 0.005418938 |
| SNORD94 | 5.37E-12 | -2.007141474 | 5.06E-13 |
| IGHV3-64 | 1.07E-10 | -1.995877886 | 1.33E-11 |
| SNORA73B | 1.43E-06 | -1.989785435 | 3.87E-07 |
| IGKV6D-21 | 1.12E-14 | -1.9788863 | 6.09E-16 |
| MS4A1 | 3.65E-16 | -1.907747938 | 1.41E-17 |
| SAA2 | 3.84E-24 | -1.880935878 | 1.90E-26 |
| PTN | 5.50E-19 | -1.864637443 | 1.12E-20 |
| IGLVI-70 | 6.70E-09 | -1.839916644 | 1.17E-09 |
| PLA2G2A | 4.68E-09 | -1.824030195 | 7.91E-10 |
| SCGB1B2P | 9.15E-09 | -1.811337001 | 1.63E-09 |
| SNORA5A | 0.021335338 | -1.782255053 | 0.013517671 |
| SAA2-SAA4 | 5.10E-27 | -1.761341568 | 1.11E-29 |
| SCARNA12 | 0.034968371 | -1.752704715 | 0.023374565 |
| SAA1 | 7.75E-28 | -1.743905992 | 1.26E-30 |
| MTRNR2L8 | 0.01696556 | -1.738094744 | 0.010486151 |
| CR2 | 3.23E-12 | -1.726996766 | 2.93E-13 |
| LEP | 1.18E-13 | -1.718631804 | 8.06E-15 |
| CIDEA | 1.07E-14 | -1.699659199 | 5.83E-16 |
| IGKV2D-24 | 1.17E-12 | -1.6975783 | 9.75E-14 |
| CIDEC | 3.70E-17 | -1.687386454 | 1.15E-18 |
| CXCL2 | 3.17E-35 | -1.686416227 | 4.91E-39 |
| IGHV3-72 | 3.92E-18 | -1.668704496 | 9.76E-20 |
| VMO1 | 0.004763217 | -1.664366606 | 0.002600467 |
| IGKV1D-39 | 5.83E-11 | -1.627732258 | 6.86E-12 |
| IGLV4-3 | 3.31E-08 | -1.611958804 | 6.59E-09 |
| IGKV6-21 | 6.49E-17 | -1.611658897 | 2.12E-18 |
| PI3 | 4.99E-11 | -1.60214983 | 5.80E-12 |
| C7 | 1.08E-22 | -1.586709001 | 7.85E-25 |
| RN7SL674P | 0.000327399 | -1.583901847 | 0.000140082 |
| PLIN4 | 6.16E-17 | -1.579344851 | 2.00E-18 |
| CCL19 | 1.17E-24 | -1.578096729 | 5.08E-27 |
| PLIN1 | 4.40E-15 | -1.558439391 | 2.21E-16 |
| C6orf15 | 0.000508429 | -1.549015687 | 0.000226323 |
| IGLV7-43 | 8.59E-13 | -1.536753157 | 7.01E-14 |
| LTB | 5.51E-16 | -1.523885355 | 2.21E-17 |
| GPD1 | 4.43E-15 | -1.517996301 | 2.23E-16 |
| FOSB | 1.01E-17 | -1.507831563 | 2.85E-19 |
| IGHJ3 | 3.64E-12 | -1.504225514 | 3.33E-13 |
| IGHJ3P | 1.93E-13 | -1.504154052 | 1.37E-14 |
| ACKR1 | 2.01E-28 | -1.501753554 | 2.02E-31 |
| ADIPOQ | 2.24E-12 | -1.495825906 | 1.97E-13 |
| CHIT1 | 9.20E-06 | -1.488952684 | 2.91E-06 |
| ARHGAP40 | 1.16E-14 | -1.483425996 | 6.31E-16 |
| IGHD | 3.71E-19 | -1.482715696 | 7.28E-21 |
| CD52 | 3.02E-14 | -1.47746576 | 1.80E-15 |
| CAVIN2 | 9.69E-29 | -1.475432768 | 8.26E-32 |
| MT1M | 9.44E-24 | -1.46128423 | 5.41E-26 |
| PIGR | 1.96E-14 | -1.458480371 | 1.12E-15 |
| CXCL13 | 1.38E-09 | -1.455878471 | 2.12E-10 |
| ADH1B | 5.94E-17 | -1.45075631 | 1.93E-18 |
| XDH | 3.93E-05 | -1.447100673 | 1.40E-05 |
| CXCL1 | 4.01E-16 | -1.441456572 | 1.55E-17 |
| LGALS7B | 5.85E-05 | -1.437566564 | 2.16E-05 |
| IGHV1OR15-2 | 2.75E-10 | -1.436197994 | 3.68E-11 |
| TRARG1 | 4.65E-14 | -1.417609228 | 2.91E-15 |
| SPIB | 1.19E-15 | -1.407780041 | 5.22E-17 |
| VPREB3 | 2.71E-11 | -1.401508966 | 2.97E-12 |
| RBP4 | 1.23E-15 | -1.380470993 | 5.41E-17 |
| IGHV3-13 | 3.05E-11 | -1.378412173 | 3.38E-12 |
| FABP4 | 7.74E-14 | -1.370587939 | 5.08E-15 |
| PI16 | 5.69E-22 | -1.356932229 | 5.07E-24 |
| AC024940.1 | 3.15E-10 | -1.323127876 | 4.28E-11 |
| IGLV2-14 | 4.49E-11 | -1.320537821 | 5.19E-12 |
| OXTR | 1.79E-11 | -1.318337763 | 1.89E-12 |
| IGKV1OR2-6 | 1.38E-11 | -1.310233925 | 1.43E-12 |
| IGKV2D-40 | 9.69E-10 | -1.308597405 | 1.44E-10 |
| IGLV9-49 | 1.88E-06 | -1.308420288 | 5.20E-07 |
| IL6 | 7.24E-17 | -1.307708357 | 2.40E-18 |
| IGLC6 | 3.16E-13 | -1.305200399 | 2.39E-14 |
| PDK4 | 2.01E-22 | -1.303825382 | 1.59E-24 |
| CD79A | 2.96E-15 | -1.303541073 | 1.43E-16 |
| CD79B | 1.74E-25 | -1.30127444 | 5.54E-28 |
| CHRDL1 | 1.23E-20 | -1.30027483 | 1.65E-22 |
| IGHV1-24 | 3.26E-10 | -1.298087273 | 4.43E-11 |
| TESC | 9.02E-26 | -1.287472864 | 2.59E-28 |
| SCARNA9 | 0.040759519 | -1.285129646 | 0.02768411 |
| COL17A1 | 3.81E-20 | -1.284613737 | 5.78E-22 |
| MYH11 | 4.01E-17 | -1.27780092 | 1.26E-18 |
| KCNIP2 | 1.10E-20 | -1.271419772 | 1.44E-22 |
| MMRN1 | 8.55E-19 | -1.270129484 | 1.86E-20 |
| SNORA31 | 3.87E-14 | -1.26970769 | 2.35E-15 |
| IL33 | 3.52E-32 | -1.266226873 | 8.17E-36 |
| OLFM4 | 7.21E-13 | -1.25865151 | 5.83E-14 |
| IGLV2-18 | 4.25E-16 | -1.257349101 | 1.65E-17 |
| MYBPC1 | 1.75E-14 | -1.256598498 | 9.83E-16 |
| SNORA13 | 0.000150849 | -1.255075293 | 6.03E-05 |
| CD207 | 2.36E-11 | -1.237042072 | 2.55E-12 |
| IGLV7-46 | 6.41E-14 | -1.235795342 | 4.15E-15 |
| IGLV2-11 | 1.05E-12 | -1.233161004 | 8.68E-14 |
| IGHV3-43 | 1.41E-11 | -1.231052783 | 1.46E-12 |
| TNXB | 5.69E-21 | -1.219561767 | 6.56E-23 |
| MEOX1 | 4.28E-28 | -1.218776727 | 6.30E-31 |
| CD1C | 3.69E-24 | -1.218238035 | 1.77E-26 |
| SNORA71A | 3.34E-05 | -1.217919483 | 1.18E-05 |
| FMO2 | 8.07E-21 | -1.217299509 | 9.90E-23 |
| TCN1 | 2.23E-08 | -1.213432573 | 4.30E-09 |
| IGKV1D-17 | 1.16E-09 | -1.211460948 | 1.75E-10 |
| GPIHBP1 | 3.18E-18 | -1.206294387 | 7.61E-20 |
| G0S2 | 1.30E-15 | -1.197998423 | 5.75E-17 |
| SLC28A3 | 1.15E-15 | -1.197033878 | 5.02E-17 |
| IGLV3-25 | 7.61E-08 | -1.196712203 | 1.63E-08 |
| IGHA2 | 2.08E-23 | -1.194387057 | 1.34E-25 |
| TRBJ2-3 | 1.03E-13 | -1.193096722 | 6.96E-15 |
| NGFR | 2.44E-26 | -1.190331961 | 6.06E-29 |
| ACY3 | 3.70E-09 | -1.189573453 | 6.16E-10 |
| ITIH5 | 3.90E-24 | -1.188913199 | 1.96E-26 |
| IGHM | 3.54E-14 | -1.188049019 | 2.14E-15 |
| CCL21 | 1.08E-18 | -1.183893464 | 2.37E-20 |
| TP63 | 5.39E-20 | -1.178078152 | 8.44E-22 |
| IL7R | 2.77E-17 | -1.173069193 | 8.38E-19 |
| FOS | 3.14E-20 | -1.173049024 | 4.72E-22 |
| IGHV3-71 | 2.50E-12 | -1.167353715 | 2.22E-13 |
| MEOX2 | 6.54E-21 | -1.1617464 | 7.75E-23 |
| IGLV4-69 | 2.01E-09 | -1.160249884 | 3.16E-10 |
| ROCR | 1.20E-20 | -1.158449203 | 1.60E-22 |
| ZAP70 | 3.69E-18 | -1.155609736 | 9.06E-20 |
| LCN2 | 5.83E-07 | -1.155337603 | 1.46E-07 |
| HSPB6 | 3.36E-21 | -1.15263061 | 3.59E-23 |
| C2orf40 | 1.12E-23 | -1.141695558 | 6.53E-26 |
| MEG3 | 5.39E-15 | -1.138626505 | 2.76E-16 |
| AL691482.3 | 3.35E-12 | -1.138243123 | 3.05E-13 |
| POU2AF1 | 8.94E-16 | -1.138071285 | 3.81E-17 |
| PLA2G2D | 4.89E-09 | -1.137844156 | 8.30E-10 |
| IGKV1-9 | 2.39E-13 | -1.132944526 | 1.75E-14 |
| SERPINF2 | 2.01E-27 | -1.132025721 | 3.58E-30 |
| TSHZ2 | 2.89E-22 | -1.120876957 | 2.48E-24 |
| CHI3L1 | 5.45E-11 | -1.112987432 | 6.38E-12 |
| CLEC10A | 1.20E-21 | -1.111932667 | 1.17E-23 |
| SNORA26 | 1.40E-06 | -1.108931193 | 3.77E-07 |
| GZMM | 2.06E-18 | -1.107252829 | 4.77E-20 |
| EGR1 | 1.06E-20 | -1.103484155 | 1.38E-22 |
| IGHV1-45 | 1.99E-10 | -1.10226376 | 2.60E-11 |
| IGLV3-19 | 2.88E-08 | -1.101334945 | 5.67E-09 |
| OR2I1P | 1.86E-09 | -1.100101298 | 2.92E-10 |
| FCER1A | 1.03E-20 | -1.096811931 | 1.32E-22 |
| SELP | 4.46E-24 | -1.093397035 | 2.28E-26 |
| ITGA7 | 7.47E-27 | -1.089044271 | 1.68E-29 |
| TRBJ2-7 | 2.35E-10 | -1.084912591 | 3.11E-11 |
| MFAP4 | 2.23E-25 | -1.084277747 | 7.59E-28 |
| LIPE | 2.27E-11 | -1.083552504 | 2.44E-12 |
| GPR171 | 9.44E-17 | -1.081197879 | 3.18E-18 |
| NR4A1 | 5.57E-16 | -1.080055607 | 2.25E-17 |
| IGHV1-18 | 1.77E-07 | -1.077513905 | 4.04E-08 |
| ZFP36 | 1.38E-30 | -1.07416162 | 6.42E-34 |
| CD69 | 8.17E-22 | -1.069201579 | 7.52E-24 |
| CD1E | 1.15E-18 | -1.06717082 | 2.53E-20 |
| CTSG | 6.26E-15 | -1.065804659 | 3.24E-16 |
| IGKV1-5 | 6.72E-11 | -1.063612848 | 8.04E-12 |
| IGKV1D-12 | 2.96E-08 | -1.059696606 | 5.83E-09 |
| IGLV3-16 | 8.69E-12 | -1.057752953 | 8.67E-13 |
| IGKV2-24 | 2.20E-15 | -1.053917441 | 1.01E-16 |
| JCHAIN | 2.52E-21 | -1.053152836 | 2.64E-23 |
| CLDN5 | 3.79E-24 | -1.051902829 | 1.85E-26 |
| S100B | 2.62E-27 | -1.051168486 | 5.07E-30 |
| RRAD | 3.03E-23 | -1.049612484 | 2.02E-25 |
| BTNL9 | 1.10E-19 | -1.045167517 | 1.92E-21 |
| IGKV3OR2-268 | 3.57E-13 | -1.043143783 | 2.72E-14 |
| CNN1 | 1.16E-15 | -1.03738454 | 5.06E-17 |
| ITM2A | 4.43E-28 | -1.036706265 | 6.87E-31 |
| PTGDS | 1.17E-21 | -1.036617558 | 1.14E-23 |
| GZMK | 6.72E-16 | -1.030795317 | 2.79E-17 |
| HLF | 1.16E-18 | -1.030329743 | 2.57E-20 |
| S1PR4 | 2.31E-17 | -1.029997282 | 6.91E-19 |
| NR4A3 | 2.01E-12 | -1.029117305 | 1.75E-13 |
| CCL17 | 1.25E-11 | -1.027998202 | 1.28E-12 |
| SYT8 | 1.70E-17 | -1.026201534 | 4.91E-19 |
| TRBV29-1 | 1.35E-13 | -1.022302885 | 9.31E-15 |
| AREG | 1.76E-09 | -1.022133077 | 2.75E-10 |
| RHCG | 5.91E-05 | -1.02072482 | 2.19E-05 |
| NFKBIZ | 5.49E-20 | -1.019314255 | 8.63E-22 |
| IGKV3-7 | 4.04E-14 | -1.012486234 | 2.48E-15 |
| SNORA55 | 0.002453026 | -1.007966561 | 0.001253883 |
| INMT | 1.96E-22 | -1.007477287 | 1.52E-24 |
| FAM110D | 7.92E-25 | -1.005858337 | 3.20E-27 |
| PER1 | 3.69E-28 | -1.00265068 | 5.14E-31 |
| AC015911.7 | 2.94E-17 | -1.001539444 | 8.93E-19 |
| PSCA | 0.009837645 | 1.023226177 | 0.005763399 |
| LINC00052 | 0.014027789 | 1.04465053 | 0.008517105 |
| SLC9A2 | 0.001026434 | 1.065909205 | 0.000484506 |
| CEACAM6 | 1.20E-08 | 1.067731362 | 2.19E-09 |
| CEACAM5 | 7.89E-11 | 1.074654871 | 9.58E-12 |
| HHIPL2 | 2.33E-08 | 1.07762777 | 4.52E-09 |
| SLC27A2 | 0.000899145 | 1.087516549 | 0.000420103 |
| SYT13 | 3.08E-07 | 1.102285103 | 7.34E-08 |
| LINC02224 | 0.005930199 | 1.140645985 | 0.003304204 |
| RNU6-813P | 1.34E-05 | 1.168359274 | 4.38E-06 |
| ASCL1 | 0.00051301 | 1.174420054 | 0.000228521 |
| AL161646.1 | 0.004196441 | 1.216445203 | 0.00225705 |
| WT1 | 0.002698447 | 1.366156422 | 0.001393549 |
| MUC2 | 0.00053227 | 1.622544225 | 0.000238132 |
| LINC00578 | 0.019703348 | 1.72900706 | 0.012392072 |
| FAM25A | 0.008691803 | 1.788127231 | 0.005030821 |
| CPLX2 | 1.85E-05 | 1.89375845 | 6.18E-06 |
| BPIFB2 | 0.003090651 | 1.923028407 | 0.001610941 |
| CGA | 1.71E-05 | 2.041958962 | 5.67E-06 |
| CRISP3 | 0.000229052 | 2.085022936 | 9.50E-05 |
| SIRLNT | 2.69E-10 | 2.176054593 | 3.60E-11 |
| AC026355.1 | 0.000649851 | 2.336886211 | 0.000295771 |
| SPDYC | 2.96E-06 | 2.642817167 | 8.49E-07 |
| NSG2 | 0.019221773 | 2.940668141 | 0.01205047 |

Abbreviations: AHGs, apoptosis and hypoxia-related genes; Log2 FC, Log2 fold change; FDR, false discovery rate.
